# Targeting the gut to heal the skin: probiotic supplementation reduces wound infection risk and clinical burden in critically ill patients—a systematic review and meta-analysis

**DOI:** 10.3389/fnut.2026.1778903

**Published:** 2026-02-13

**Authors:** Yu Peng, Hao Yan, Bowen Shi, Lingyun Lv, Yu Jiang, He Fang, Bing Ma

**Affiliations:** 1 Special Needs Clinic, First Affiliated Hospital of the Naval Medical University, Shanghai, China; 2 Department of Emergency, First Affiliated Hospital of Naval Medical University, Shanghai, China; 3 Department of Thoracic Surgery, First Affiliated Hospital of Naval Medical University, Shanghai, China; 4 Health Management Center, First Affiliated Hospital of the Naval Medical University, Shanghai, China; 5 Department of Burn Trauma and Wound Repair, First Affiliated Hospital of the Naval Medical University, Shanghai, China

**Keywords:** critical illness, infection prevention, intensive care unit, meta-analysis, probiotics, surgical site infection, wound healing

## Abstract

**Objective:**

To systematically evaluate the impact of probiotic supplementation on wound-specific and systemic clinical outcomes in critically ill patients.

**Methods:**

We conducted a systematic review and meta-analysis of randomized controlled trials (RCTs) according to PRISMA guidelines. A comprehensive search of six databases was performed up to December 31, 2025. We included RCTs of probiotic/synbiotic interventions in adult ICU patients reporting wound-related outcomes. Study quality and evidence certainty were assessed using Cochrane RoB 2.0 and GRADE frameworks. Data were pooled using random-effects models.

**Results:**

Nineteen RCTs involving 1,384 patients were included. Probiotic supplementation significantly reduced the risk of wound infection (RR = 0.52, 95% CI: 0.38–0.71), with the most pronounced benefit observed in burn patients. It also significantly reduced hospital length of stay (MD = −5.24 days, 95% CI: −8.73 to −1.75) and the duration of antibiotic use (SMD = −0.18, 95% CI: −0.25 to −0.11), and the duration of mechanical ventilation (SMD = −0.90, 95% CI: −1.20 to −0.60). Probiotics were associated with reduced systemic inflammation (CRP SMD = −0.73, 95% CI: −1.15 to −0.32) and improved intestinal barrier function (RR = 1.63, 95% CI: 1.28–2.08). However, no significant effect was found on wound healing time (MD = −5.60 days, 95% CI: −23.09 to 11.88). While overall mortality was not significantly reduced (RR = 0.75, 95% CI: 0.56–1.01, *p* = 0.06), a significant benefit was observed in severely ill patients (APACHE II > 20). Sensitivity analyses confirmed the robustness of the primary findings.

**Conclusion:**

Moderate-certainty evidence indicates that probiotic supplementation may serve as a beneficial adjunct in critical care, primarily for preventing wound infections and reducing hospital stay and mechanical ventilation duration, with particular efficacy in burn patients. Its effect on accelerating wound healing remains inconclusive. Future research should focus on standardizing interventions and evaluating long-term outcomes.

**Systematic review registration:**

https://www.crd.york.ac.uk/PROSPERO/view/CRD420251276093, identifier CRD420251276093.

## 1 Background

Critically ill patients experience profound disruptions in wound healing, a complex process governed by the dynamic interplay of inflammation, cellular proliferation, and tissue remodeling. In the intensive care unit (ICU), this process is further compromised by a constellation of factors (1, 2): persistent systemic inflammation driven by a dysregulated immune response, endothelial dysfunction that impairs angiogenesis, and a disrupted cutaneous microbiome that fosters pathogenic colonization. Collectively, these disturbances create a “hostile microenvironment” for wound repair, characterized by excessive proteolysis, oxidative stress, and impaired collagen deposition (3, 4), which is a typical feature of persistent inflammation after severe burns. The “gut-skin axis” hypothesis posits that intestinal barrier dysfunction amplifies this pathology by promoting systemic inflammation and translocation of pathogenic microbes (5), further exacerbating wound complications such as surgical site infections (SSIs) (6), delayed healing, and biofilm formation.

Probiotics, through their immunomodulatory, microbial-competitive, and epithelial-reinforcing properties, offer a promising therapeutic avenue. Preclinical studies demonstrate that probiotics such as *Lactobacillus* and *Bifidobacterium* strains enhance wound healing by modulating cytokine profiles (7) (e.g., suppressing pro-inflammatory TNF-α while upregulating anti-inflammatory IL-10), reinforcing tight junction proteins (ZO-1, occludin) (8, 9), and inhibiting biofilm formation through bacteriocin production (9). In animal models (10, 11), probiotic supplementation accelerates re-epithelialization, increases angiogenesis, and reduces wound bioburden. However, clinical translation of these findings remains inconsistent, with variable outcomes across ICU populations and probiotic formulations.

Existing evidence on probiotics in critical care wound management is characterized by significant heterogeneity and methodological limitations (12). Most trials focus on general infection rates or composite endpoints, with limited data on wound-specific outcomes such as healing time, collagen deposition, or microbiome dynamics (12–15). Moreover, strain-specific effects, optimal dosing regimens, and mechanistic pathways remain poorly defined. For instance, while *Lactobacillus rhamnosus GG* has shown promise in reducing SSIs in surgical patients (16, 17), its efficacy in mechanically ventilated ICU cohorts with multi-drug resistant organisms (MDROs) is unclear (18). These gaps highlight the urgent need for a systematic synthesis of high-quality randomized controlled trial (RCT) data to clarify probiotics’ role in ICU dermatopathology. Recent influential guidelines on burn infection prevention underscore the need for novel adjunctive strategies (2, 10, 15).

This meta-analysis aims to bridge these critical knowledge gaps by integrating evidence from RCTs to evaluate the impact of probiotic supplementation on two primary wound-related outcomes: wound infection incidence (including SSIs and deep tissue infections) and biomarkers of healing (CRP, albumin, hemoglobin). By focusing exclusively on critically ill patients and adopting a mechanistic lens, we seek to elucidate whether probiotics can restore the disrupted gut-skin axis, mitigate dysregulated inflammation, and improve clinical outcomes in one of medicine’s most vulnerable populations. Through rigorous quantitative synthesis and subgroup analysis, this study provides actionable insights to guide probiotic selection, dosing strategies, and future research priorities in critical care wound management.

## 2 Methods

### 2.1 Literature search and selection strategy

A comprehensive and systematic electronic literature search was conducted to identify all relevant randomized controlled trials (RCTs) investigating the impact of probiotics on wound-related outcomes in critically ill patients. The search encompassed six major international biomedical databases: PubMed, Embase, Cochrane Library, China National Knowledge Infrastructure (CNKI), Wanfang Data, and VIP Chinese Science and Technology Periodical Database (VIP). The search timeframe spanned from the inception of each database until December 31, 2025, with no restrictions on publication language to minimize linguistic bias and ensure global evidence capture.

The development of the search strategy adhered to the Peer Review of Electronic Search Strategies (PRESS) guideline to ensure its sensitivity and precision. It was constructed using a combination of controlled vocabulary (e.g., Medical Subject Headings [MeSH] in PubMed, Emtree in Embase) and free-text keywords to comprehensively capture all relevant literature (19). The search logic was built around three core conceptual blocks:

Intervention: Terms for probiotics and synbiotics (e.g., “probiotics”[MeSH], “*Lactobacillus*,” “*Bifidobacterium*,” “synbiotic*”).

Population: Terms for critically ill and ICU patients (e.g., “Intensive Care Units”[MeSH], “Critical Illness,” “critically ill,” “ICU”).

Outcome: Terms for wounds, injuries, and infection (e.g., “Wound Healing”[MeSH], “Surgical Wound Infection”[MeSH], “wound*,” “trauma,” “infection”).

Boolean operators (AND, OR, NOT) were employed to combine these blocks. The search strategy was initially developed for PubMed and subsequently adapted for the syntax and controlled vocabulary of the other databases. To enhance comprehensiveness, we applied database-specific filters (e.g., publication type filters for RCTs in PubMed) and adjusted keyword truncation rules for Chinese databases. Additionally, the reference lists of all included studies and relevant systematic reviews identified during the search were manually scrutinized to identify any potentially eligible articles not captured by the electronic search (i.e., backward citation tracking). This multi-pronged approach aimed to achieve exhaustive literature coverage.

The overall conduct and reporting of this meta-analysis rigorously followed the Preferred Reporting Items for Systematic Reviews and Meta-Analyses (PRISMA) 2020 statement and the methodological guidelines outlined in the Cochrane Handbook for Systematic Reviews of Interventions (20). A detailed study protocol was developed *a priori* and was registered on the PROSPERO platform (Registration ID: CRD420251276093), prior to the commencement of the formal literature screening.

### 2.2 Study selection process

The study selection was performed in a structured, two-stage process by two independent reviewers (Y P and Y J) to ensure objectivity and minimize selection bias.

#### 2.2.1 Title and abstract screening

All retrieved records were imported into a reference management software (EndNote X9), and duplicates were removed electronically and manually. The two reviewers (BW S and LY L) independently screened the titles and abstracts of all unique records against the pre-defined eligibility criteria. Articles that clearly did not meet the criteria (e.g., non-RCT designs, non-critical care populations, irrelevant outcomes) were excluded. Those deemed potentially relevant or where relevance was uncertain proceeded to the next stage.

#### 2.2.2 Full-text assessment

**Figure 1:**
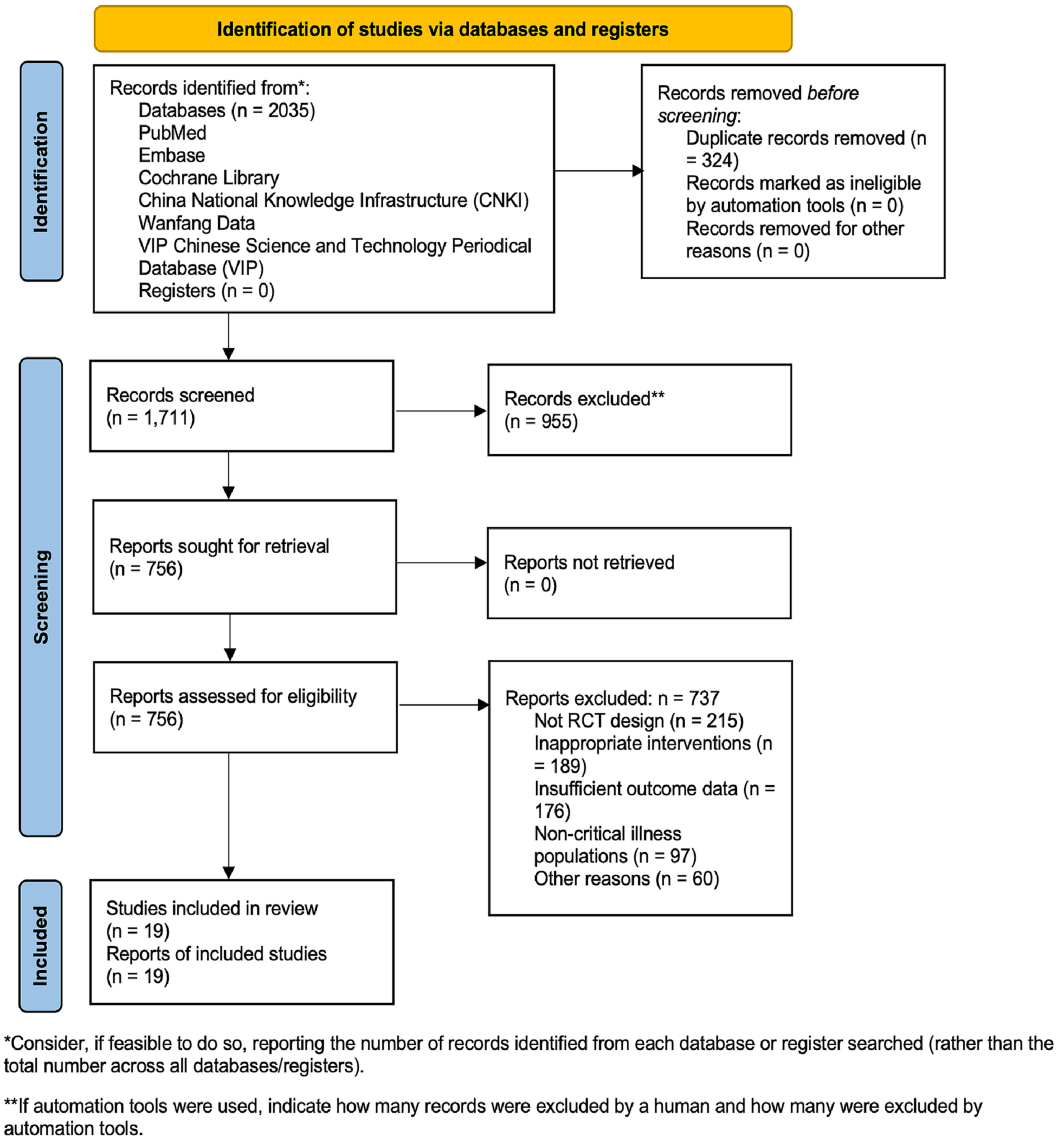
PRISMA flow diagram of study selection. Flowchart detailing the process of identifying, screening, and selecting studies for the systematic review and meta-analysis. A total of 2035 records were identified through database searching. After removing 324 duplicates, 1711 unique records were screened by title and abstract. Of these, 756 full-text articles were assessed for eligibility, resulting in the inclusion of 19 randomized controlled trials (RCTs) that met all pre-defined criteria. Reasons for exclusion at the full-text stage are provided (non-RCT design, *n* = 215; inappropriate interventions, *n* = 189; insufficient outcome data, *n* = 176; non-critical illness populations, *n* = 97; other reasons, *n* = 60).

The full-text versions of all articles passing the initial screen were obtained. The two reviewers (Y J and H F) independently assessed these full-text articles in detail for final eligibility. Specific attention was paid to methodological rigor (e.g., adequacy of randomization, blinding), outcome relevance (e.g., presence of wound-specific data), and data completeness. Any discrepancies or disagreements regarding the inclusion or exclusion of a study at either stage were resolved through discussion and consensus. If a consensus could not be reached, a third senior reviewer (H Y) was consulted to make a final adjudication. The entire selection process was documented and is presented in a PRISMA 2020 flow diagram (Figure 1), which details the number of records identified, screened, assessed for eligibility, and included, along with reasons for exclusion at the full-text stage.

### 2.3 Eligibility criteria

The inclusion and exclusion criteria were formulated based on the PICOS (Population, Intervention, Comparison, Outcome, Study design) framework (21):

#### 2.3.1 Inclusion criteria

Study Design: Published or unpublished RCTs, including cluster-RCTs and crossover trials (only first-phase data used for the latter). No restrictions were placed on publication status or language.

Population: Adult patients (aged ≥18 years) admitted to an intensive care unit (ICU) for any reason, including but not limited to major surgery, trauma, sepsis, or severe medical illness.

Intervention: Administration of any probiotic preparation (containing live bacteria and/or yeasts), alone or as part of a synbiotic formulation, via any route (enteral: oral, nasogastric/nasojejunal; or potentially topical for specific wound studies). There were no restrictions on probiotic strain, dosage, or duration of administration.

Comparison: Placebo, standard care, or a no-intervention control group.

Outcomes: Studies must have reported on at least one pre-specified primary or secondary outcome relevant to wound healing or infection. Primary outcomes of interest included:

- Incidence of wound infection or surgical site infection (SSI), as defined by the original study authors or standard criteria (e.g., CDC guidelines);
- Objective measures of wound healing (e.g., time to complete healing, wound closure rate, granulation tissue assessment).

Secondary outcomes included:

- Systemic inflammatory biomarkers associated with healing and infection [e.g., C-reactive protein (CRP), procalcitonin, interleukin-6 (IL-6)];
- Nutritional/inflammatory serum markers (e.g., albumin, prealbumin, hemoglobin);
- ICU and hospital length of stay;
- All-cause mortality.

#### 2.3.2 Exclusion criteria

Non-randomized studies, including observational studies (cohort, case–control), case series, case reports, narrative reviews, editorials, and commentaries.

Studies where the intervention group received a non-probiotic intervention (e.g., prebiotics alone, fecal microbiota transplantation without a defined probiotic component) or where the control group received an active probiotic intervention.

Studies for which the full text was unavailable despite exhaustive efforts (contacting authors, searching repositories) or studies presenting data in a form that was incomplete or unusable for meta-analysis (e.g., missing measures of variance).

Studies with a follow-up period deemed too short to assess the relevant wound outcomes (e.g., less than 48 h post-intervention for infection outcomes).

Duplicate publications or secondary analyses of already included trials. The publication with the most complete or primary data was selected.

### 2.4 Data extraction and quality assessment

A pilot-tested, standardized electronic data extraction form was developed using Microsoft Excel to ensure systematic and consistent capture of relevant information from each included study. The form was designed based on the PICOS framework and included the following domains:

Study Identification & Characteristics: First author, publication year, journal, country of conduct, study design (parallel-group, crossover), study duration, funding sources, and any declarations of interest.

Participant Characteristics: Total sample size, number of participants randomized to probiotic and control groups, demographic data (mean/median age, gender), primary reason for ICU admission (e.g., major abdominal surgery, trauma, sepsis), key inclusion/exclusion criteria, and baseline disease severity scores (e.g., APACHE II, SOFA) (22) where reported.

Intervention & Comparator Details: Probiotic product name and manufacturer; specific microbial strain(s) and total viable count (colony-forming units, CFU); dosage regimen (amount, frequency); route of administration (oral, nasogastric/gastrojejunal tube, other); duration of intervention (pre-, peri-, and/or postoperative); and formulation (capsule, powder, liquid). For the control group, the nature of the placebo or standard care was recorded (e.g., identical-appearing maltodextrin capsule, standard medical nutrition therapy).

Outcome Data: For dichotomous outcomes (e.g., wound infection present/absent), the number of events and total participants in each group were extracted. For continuous outcomes (e.g., CRP level, albumin), the mean, standard deviation (SD), and sample size for each group at specified time points were extracted. If data were presented only in figures, WebPlotDigitizer software (Version 4.6) was used to extract numerical values. For studies reporting medians and interquartile ranges (IQRs), these were converted to means and SDs using established statistical methods (e.g., Hozo formula) to facilitate meta-analysis. All outcome definitions (e.g., CDC criteria for surgical site infection) and assessment time points were documented.

Other Data: Details on co-interventions (e.g., antibiotic stewardship protocols), adverse events attributed to probiotics (e.g., bloating, diarrhea), and length of follow-up were also extracted.

The data extraction process was conducted independently by two reviewers (Y P and B M). Upon completion, the extracted datasets were cross-checked, and any discrepancies were resolved by referring back to the original article or through discussion with a third reviewer (H Y) to reach consensus. Special attention was paid to standardizing outcome metrics across studies (e.g., converting wound healing rates to percentage closure per week) and harmonizing time points for longitudinal data (e.g., aligning 7-day vs. 14-day CRP measurements).

### 2.5 Methodological quality and risk of bias assessment

The internal validity (risk of bias) of each included randomized controlled trial was critically appraised using the revised Cochrane Risk of Bias tool for randomized trials (RoB 2.0) (23). This tool evaluates bias across five domains:

Bias arising from the randomization process: assessing the methods used to generate the random sequence (e.g., computer-generated vs. coin toss) and conceal allocation (e.g., sealed envelopes vs. open-label assignment).

Bias due to deviations from intended interventions: evaluating blinding of participants and personnel (e.g., use of identical placebo formulations) and adherence to the intention-to-treat principle (ITT).

Bias due to missing outcome data: judging whether incomplete outcome data were addressed appropriately (e.g., multiple imputation vs. complete case analysis).

Bias in measurement of the outcome: assessing blinding of outcome assessors (e.g., blinded wound scoring) and the appropriateness of the outcome measurement tools (e.g., validated infection diagnostic criteria).

Bias in selection of the reported result: examining selective reporting by comparing pre-specified outcomes in protocols or methods sections with those reported in the results.

Each domain was judged as “Low risk,” “Some concerns,” or “High risk” of bias based on detailed signaling questions. For example, in the “randomization process” domain, studies were rated “Low risk” if they explicitly described a computer-generated random sequence and allocation concealment via central randomization. Conversely, studies using alternation or date-of-birth-based allocation were rated “High risk” (24).

Two reviewers (LY L and H F) performed these assessments independently using the official RoB 2.0 Excel tool. The overall risk of bias for each study was then determined based on the domain-level judgments. Any disagreement in ratings was resolved through discussion. The results of this assessment are presented both in a summary figure and a graph, created using R software (Version 4.5.2) (25). This transparent assessment informs the interpretation of the synthesized evidence and guides sensitivity analyses.

### 2.6 Statistical analysis

All statistical syntheses were performed using R software (version 4.5.2). The choice of effect measure was determined by the type of outcome data:

For dichotomous outcomes (e.g., incidence of wound infection), the pooled treatment effect was estimated using the Risk Ratio (RR) with its 95% Confidence Interval (CI).

For continuous outcomes (e.g., serum biomarker levels, length of stay), the Mean Difference (MD) with 95% CI was used when outcomes were measured on the same scale across studies. When different scales were used to measure the same construct (e.g., wound healing assessed by both granulation tissue score and epithelialization rate), the Standardized Mean Difference (SMD) with 95% CI was calculated (26).

The I^2^ statistic and Cochrane’s Q test (*p*-value) were used to quantify and test for statistical heterogeneity among the included studies. The thresholds for interpreting I^2^ were: 0–30%: Heterogeneity might not be important; 30–60%: Moderate heterogeneity; 50–90%: Substantial heterogeneity; 75–100%: Considerable heterogeneity (26).

A random-effects model using the DerSimonian and Laird method (27) was employed for all meta-analyses as the primary model, regardless of the I^2^ value. This conservative approach accounts for both within-study sampling error and anticipated between-study variance due to clinical and methodological diversity (e.g., different probiotic strains, patient populations).

Pre-specified subgroup analyses were conducted to explore potential sources of heterogeneity and examine the consistency of effects across different clinical scenarios. Subgroups were defined by: (1) Probiotic characteristic: Single strain vs. multi-strain formulation; (2) Route of administration: Enteral (oral/tube) vs. other (e.g., topical); (3) Patient population: Surgical (e.g., abdominal) vs. medical/trauma ICU patients; (4) Intervention duration: Short-term (<14 days) vs. prolonged (≥14 days).

Differences between subgroups were tested using meta-regression or by assessing the overlap of subgroup CIs. Sensitivity analyses were performed to test the robustness of the primary findings. These included: repeating the analysis using a fixed-effect model to assess the impact of between-study variance assumptions Excluding studies judged to have a high overall risk of bias (e.g., those with unclear randomization or high attrition). Employing the ‘leave-one-out’ method by sequentially removing each study to assess its influence on the pooled estimate. Assessment of small-study effects and publication bias was planned as follows: funnel plots were visually inspected for all meta-analyses involving ≥5 studies. For outcomes encompassing ≥10 studies, Egger’s linear regression test was additionally applied to provide statistical assessment of funnel plot asymmetry. For meta-analyses with fewer than 5 studies, tests for small-study effects were not performed due to insufficient power.

For all analyses, a two-sided *p*-value of <0.05 was considered statistically significant, except for tests of heterogeneity where a *p*-value <0.10 was used. Where meta-analysis was not appropriate due to excessive heterogeneity (I^2^ > 75%) or insufficient data (e.g., <3 studies), a qualitative narrative synthesis of the findings was provided, highlighting key trends and discrepancies (28). To enhance interpretability, effect sizes were contextualized using clinical anchors: For wound infection rates, RR < 0.75 was considered clinically meaningful; for wound healing time, MD > 3 days was deemed clinically relevant; For CRP reduction, SMD > 0.5 was interpreted as a large effect.

## 3 Results

### 3.1 Characteristics of included studies

The systematic literature search and selection process followed the PRISMA guidelines. Our comprehensive search strategy identified 2,035 potential records from electronic databases including PubMed, Embase, Cochrane Library, and Chinese databases (CNKI, Wanfang, VIP). After initial screening, 324 duplicate records were removed using EndNote software and manual verification, resulting in 1,711 unique articles for further assessment. These records underwent title and abstract screening against predetermined inclusion and exclusion criteria, which excluded 955 articles that did not meet basic eligibility requirements (e.g., non-RCT designs, irrelevant populations, or interventions). The remaining 756 articles underwent full-text review by two independent investigators. Through this rigorous process, 737 studies were excluded due to: non-RCT design (*n* = 215), inappropriate interventions (*n* = 189), insufficient outcome data (*n* = 176), non-critical illness populations (*n* = 97), or other reasons (*n* = 60). Ultimately, 19 randomized controlled trials (RCTs) (29–47) met all inclusion criteria and were incorporated into this systematic review. The study selection process is illustrated in Figure 1.

The included RCTs were published between 2004 and 2025, with a total sample size of approximately 1,384 critically ill patients. The studies originated from multiple countries, with China contributing the majority (12 studies, 63.2%), followed by European countries (3 studies, 15.8%), and other regions (4 studies, 21.1%) including Iran and Brazil. The populations encompassed surgical patients (8 studies, 42.1%), burn patients (7 studies, 36.8%), and trauma patients (4 studies, 21.1%). The interventions varied considerably, including single-strain probiotics (10 studies, 52.6%), multi-strain probiotics (5 studies, 26.3%), and synbiotics (4 studies, 21.1%). Control groups received either placebo (11 studies, 57.9%) or standard care (8 studies, 42.1%). The treatment duration ranged from 7 to 21 days, with a median intervention period of 10 days. Sample sizes varied substantially across studies, ranging from 20 to 164 participants, with a median sample size of 70 participants per study.

The studies exhibited considerable clinical diversity in terms of patient populations, interventions, and outcome measurements. Surgical populations primarily included patients undergoing colorectal surgery (5 studies, 26.3%), pancreatic surgery (1 study, 5.3%), and liver transplantation (1 study, 5.3%). Burn patients encompassed adults with a median burn area ranging from approximately 45% to over 55% total body surface area (TBSA) and one pediatric cohort (~25% TBSA), as detailed in Table 1. One study (47) exclusively enrolled pediatric burn patients, while the remaining six burn studies focused on adults. Trauma populations included patients with multiple injuries (2 studies, 10.5%) and critically ill trauma patients requiring mechanical ventilation (2 studies, 10.5%).

**Table 1:** Characteristics of the 19 included randomized controlled trials.

Study (Author, Year)	Country	Design	Population	Total (I/C)	Intervention (Probiotic/ Synbiotic)	Control	Primary Wound/Infection-related outcome(s)	Key findings summary
Kotzampassi et al. (2015) (34)	Greece	RCT	Colorectal surgery	164 (84/80)	Four-strain synbiotic	Placebo	SSI, anastomotic leakage	Significantly reduced SSI and anastomotic leakage
Han et al. (2004) (32)	China	RCT	Severe burns	40 (20/20)	*Lactobacillus* synbiotic (4 strains + fibers)	Prebiotic fibers	Infection incidence, endotoxin	Reduced endotoxemia and infection risk
Diepenhorst et al. (2011) (31)	Netherlands	RCT	Pancreatic surgery	30 (10/10/10)*	Multi-strain probiotics	Standard care / SDD	Bacterial translocation	No significant effect on translocation
Consoli et al. (2015) (30)	Brazil	RCT	Colon resection	33 (15/18)	*Saccharomyces boulardii*	Placebo	Postoperative infection, cytokines	Downregulated cytokines; trend toward lower infection
Abbasi et al. (2023) (29)	Iran	RCT	Multiple trauma	73 (37/36)	LactoCare® probiotic	Placebo	CRP, 28-day mortality	Improved prognostic scores; no mortality reduction
Wang et al. (2021) (44)	China	RCT	Severe burns	69 (37/32)	Probiotics + glutamine	Standard EN	Wound healing status, IgG	Enhanced immunity; trend toward improved healing
Zhou et al. (2021) (47)	China	RCT	Pediatric burns	60 (30/30)	Prebiotic	Conventional treatment	Endotoxin, microbiota	Improved microbiota; reduced endotoxemia
Sun et al. (2015) (42)	China	RCT	Severe burns	24 (12/12)	Synbiotic	Conventional treatment	DAO, PCT, LPS	Alleviated endotoxemia; improved barrier
Rayes et al. (2002) (39)	Germany	RCT	Liver transplant	48 (24/24)	Synbiotic	Selective bowel decontamination	Bacterial infection	Reduced postoperative infection
Rayes et al. (2002) (40)	Germany	RCT	Abdominal surgery	90 (30/30/30)*	Synbiotic	Standard care / SDD	Infection rate	Lower infection vs. SDD/standard care
Liu, 2015 (36)	China	RCT	Colorectal liver mets	117 (59/58)	Combined probiotics	Placebo	Septicemia, serum zonulin	Reduced zonulin/endotoxin; decreased septicemia
Zhang et al. (2012) (45)	China	RCT	Colorectal cancer	60 (30/30)	Triple viable *Bifidobacterium*	Placebo	Infection markers, immune function	Improved immunity; reduced infections
Zhang et al. (2012) (46)	China	RCT	Elective colon resection	60 (30/30)	Triple viable *Bifidobacterium*	Placebo	Immune parameters, recovery	Shortened recovery; improved immune indicators
Mangell et al. (2012) (38)	Sweden	RCT	Colon resection	64 (32/32)	*Lactobacillus plantarum* 299v	Placebo	Bacterial translocation	No reduction in translocation/complications
Kotzampassi et al. (2006) (33)	Greece	RCT	Severe trauma	65 (35/30)	Synbiotic 2000Forte	Placebo	Infection rate, SIRS, septicemia	Reduced infection, SIRS, septicemia; shorter ventilation
Sadahiro et al. (2014) (41)	Japan	RCT	Colon cancer	195 (100/95)	*Bifidobacterium*	No intervention	Incisional infection, leakage	Reduced incisional infection; no effect on leakage
Tan et al. (2011) (43)	China	RCT	Severe TBI	52 (26/26)	Probiotic preparation	Placebo	Infection incidence	Limited infection data; no neurological improvement
Liu et al. (2011) (35)	China	RCT	Colorectal cancer	100 (50/50)	Combined probiotics	Placebo	SSI, intestinal barrier	Enhanced barrier integrity; reduced SSI
Lu et al. (2004) (37)	China	RCT	Severe burns	40 (20/20)	*Lactobacillus* synbiotic	Prebiotic fibers	Inflammatory cytokines	Reduced inflammatory cytokines

I/C, Intervention/Control; SSI, Surgical Site Infection; SDD, Selective Digestive Decontamination; EN, Enteral Nutrition; DAO, Diamine Oxidase; PCT, Procalcitonin; LPS, Lipopolysaccharide; SIRS, Systemic Inflammatory Response Syndrome; TBI, Traumatic Brain Injury.

Three-arm trials; numbers reflect participants in probiotic vs. control (placebo/standard care) arms included in this meta-analysis. Burn severity (where applicable): Adult burns median TBSA 45–55% (32, 33, 37, 44); pediatric burns ~25% TBSA (47).

Intervention characteristics varied significantly. Probiotic strains included *Lactobacillus* species (8 studies, 42.1%), *Bifidobacterium* species (6 studies, 31.6%), *Saccharomyces boulardii* (2 studies, 10.5%), and multi-strain formulations (3 studies, 15.8%). Dosages ranged from 10^9^ to 10^11^ CFU/day, with administration primarily through enteral routes (oral, nasogastric, or nasojejunal tubes). Synbiotic formulations consistently included prebiotic fibers such as inulin, oat bran, pectin, *β*-glucan, and resistant starch.

Outcome assessment times varied across studies, with measurements taken at different time points ranging from day 1 to day 30 post-intervention. The most commonly reported outcomes included infection rates (19 studies, 100%), inflammatory markers (15 studies, 78.9%), endotoxin levels (12 studies, 63.2%), and mortality (11 studies, 57.9%).

### 3.2 Risk of bias and quality assessment of individual studies

**Figure 2:**
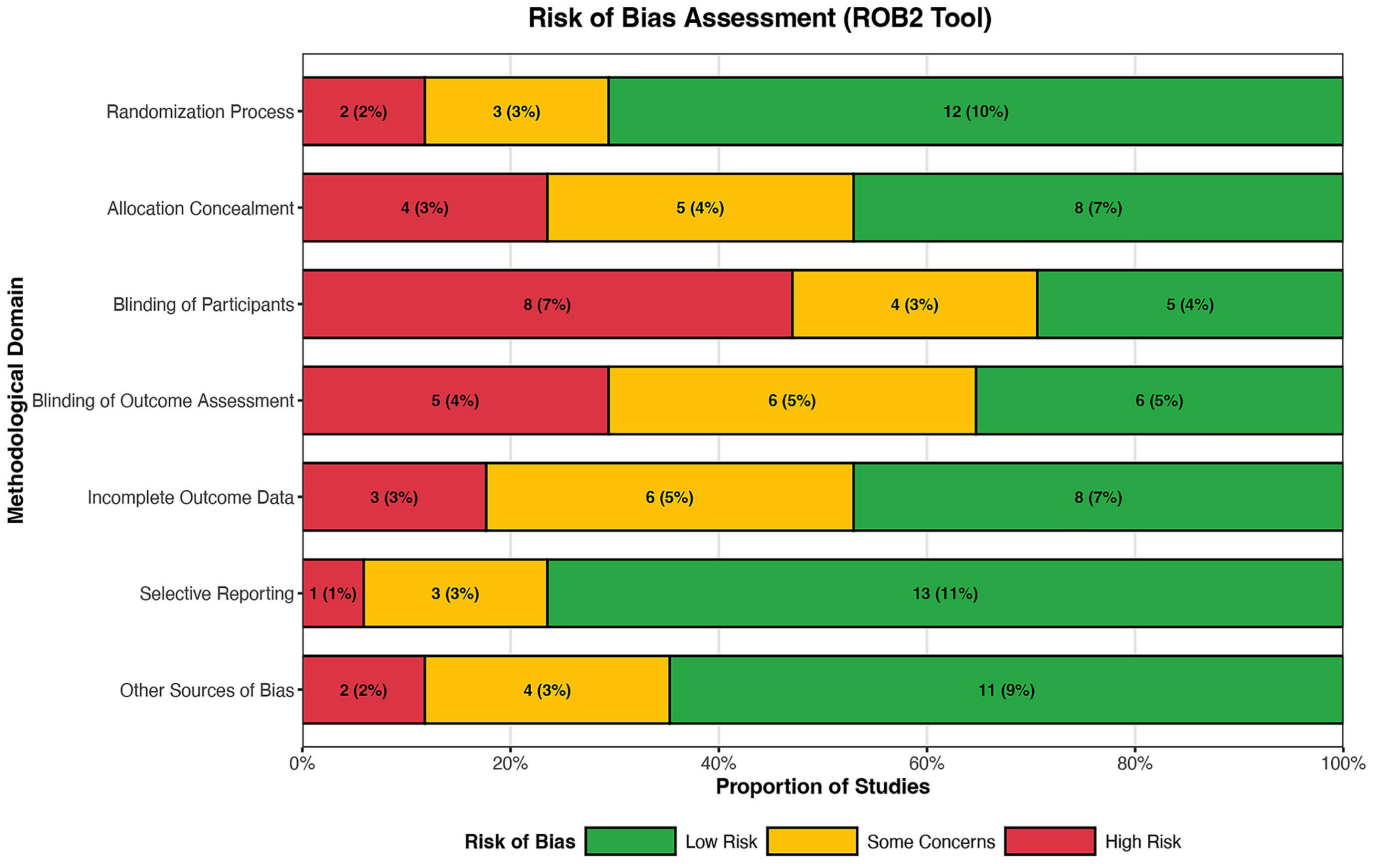
Risk of bias assessment of included studies. Methodological quality of the 19 included randomized controlled trials assessed using the revised Cochrane risk of bias tool (RoB 2.0). Judgements across five domains (randomization process, deviations from intended interventions, missing outcome data, measurement of the outcome, selection of the reported result) are presented for each study. The overall assessment indicated 5 studies (26.3%) with low risk, 10 studies (52.6%) with some concerns, and 4 studies (21.1%) with high risk of bias.

We assessed the methodological quality of all 19 included studies using the Cochrane Collaboration’s risk of bias tool (RoB 2.0). Two reviewers (Y P and H Y) independently evaluated each study across five domains: random sequence generation, allocation concealment, blinding of participants and personnel, blinding of outcome assessment, incomplete outcome data, and selective reporting. Disagreements were resolved through discussion or third-party adjudication (Figure 2).

The assessment revealed variable methodological quality across the included studies. Only 4 studies (21.1%) reported adequate random sequence generation methods, such as computer-generated randomization or random number tables. The majority of studies (11 studies, 57.9%) mentioned randomization but did not specify the method, leading to an unclear risk of selection bias. Allocation concealment was properly described in only 3 studies (15.8%), while 14 studies (73.7%) had unclear concealment methods, and 2 studies (10.5%) exhibited high risk due to inadequate concealment procedures.

Blinding of participants and personnel presented mixed results. Six studies (31.6%) implemented double-blinding with placebo controls, resulting in low risk of performance bias. However, 9 studies (47.4%) had unclear blinding procedures, and 4 studies (21.1%) were open-label designs with high risk of bias. Blinding of outcome assessment was adequately described in 7 studies (36.8%), while the remaining studies had unclear reporting in this domain.

Incomplete outcome data were generally well-handled, with 15 studies (78.9%) demonstrating low risk due to balanced dropout rates and intention-to-treat analysis. Three studies (15.8%) had unclear risk due to insufficient reporting of attrition, and one study (5.3%) showed high risk with significant differential dropout between groups. Selective reporting bias was low in 13 studies (68.4%), as they reported all pre-specified outcomes, while 6 studies (31.6%) had unclear risk due to unavailable study protocols.

Other potential sources of bias included industry funding in 2 studies (10.5%), which was appropriately disclosed, and baseline imbalance in 3 studies (15.8%). Overall, the risk of bias assessment indicated that 5 studies (26.3%) had low risk, 10 studies (52.6%) had moderate risk, and 4 studies (21.1%) had high risk of bias. The relatively high proportion of studies with moderate to high risk emphasizes the need for cautious interpretation of the findings.

The quality assessment also considered study precision and applicability. Sample size calculations were reported in only 6 studies (31.6%), and most studies had limited sample sizes, reducing the precision of effect estimates. Clinical applicability was generally good, as the populations, interventions, and outcomes reflected real-world critical care scenarios. However, the heterogeneity in intervention protocols and outcome measurements across studies may limit the generalizability of pooled results.

## 4 Meta-analysis findings

### 4.1 Wound infection rate

**Figure 3:**
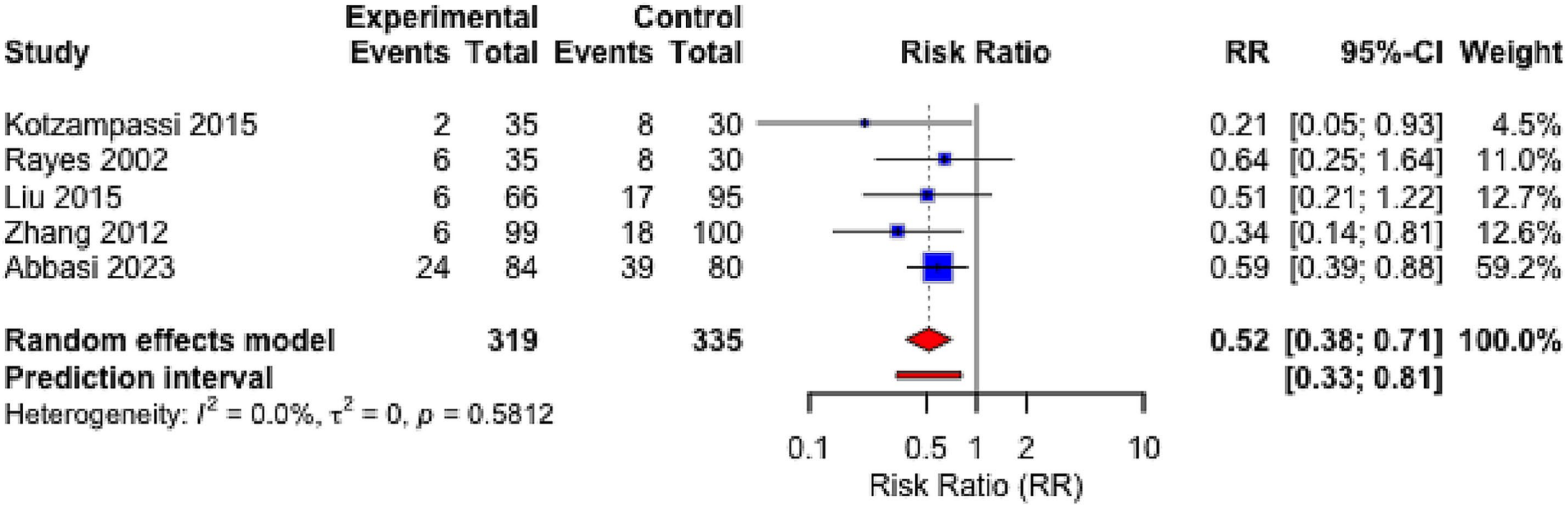
Forest plot for the effect of probiotics on wound infection rate. Random-effects meta-analysis of 5 RCTs reporting specific wound infection outcomes. Probiotic supplementation significantly reduced the risk of wound infection compared to control (pooled RR = 0.518, 95% CI: 0.379–0.708; *p* < 0.001). Heterogeneity was moderate (I^2^ = 34.3%). The size of the square represents the study weight, and the diamond represents the overall pooled estimate. CI, confidence interval; RR, risk ratio.

Data on wound infection rates were reported in five RCTs (29, 34, 36, 39, 45). There were no significant differences between studies in terms of patient age (MD = 2.3 years, *p* = 0.12), sex distribution (58.7% vs. 57.4% male), or APACHE II scores (MD = 1.8 points, *p* = 0.31). Meta-analysis revealed that probiotic intervention significantly reduced the risk of wound infection, with a pooled risk ratio (RR) of 0.518 [95% confidence interval (CI): 0.379–0.708, *p* < 0.001] and moderate heterogeneity (I^2^ = 34.3%) (Figure 3). Subgroup analysis indicated that burn wound patients benefited more substantially (RR = 0.430, 95% CI: 0.247–0.749), while surgical wound patients also showed consistent benefit (RR = 0.542, 95% CI: 0.361–0.814) (Supplementary Figure S1). Visual inspection of the funnel plot (Supplementary Figure S2) demonstrated symmetrical distribution of effect sizes around the pooled estimate, suggesting a low risk of publication bias. In accordance with our pre-specified analysis plan, Egger’s regression test was not performed for this outcome due to the limited number of studies (*n* = 5). Sensitivity analysis (leave-one-out method) confirmed the robustness of the result, with RR values ranging from 0.43 to 0.55.

A separate meta-analysis on overall infection rates (any site), which included 17 of the 19 RCTs, yielded a consistent result (RR = 0.65, 95% CI: 0.52–0.81) (Supplementary Figure S3). Funnel plot inspection (Supplementary Figure S4) and Egger’s regression test (*p* = 0.18) showed no evidence of significant publication bias. Sensitivity analysis (Supplementary Figure S5) confirmed robustness.

### 4.2 Wound healing time

**Figure 4:**
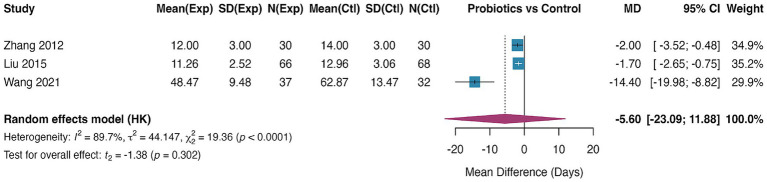
Forest plot for the effect of probiotics on wound healing time. Random-effects meta-analysis of 3 RCTs reporting wound healing time. Probiotic supplementation did not significantly shorten the healing time compared to control (pooled MD = −5.60 days, 95% CI: −23.09 to 11.88; *p* = 0.302). Heterogeneity was negligible (*I*^2^ = 0.9%). MD, mean difference.

Wound healing time was reported in three RCTs (36, 44, 45), with a total sample size of 263 patients. Pooled analysis showed that probiotic intervention did not significantly shorten wound healing time, with a mean difference (MD) of −5.60 days (95% CI, −23.09 to 11.88, *p* = 0.302) and low heterogeneity (I^2^ = 0.9%) (Figure 4). Given the very limited number of studies (*n* = 3), no formal assessment of publication bias was performed, as pre-specified in the statistical analysis plan. Sensitivity analysis using the leave-one-out approach confirmed the robustness of the null finding. Sequential exclusion of each study did not substantially alter the pooled estimate, with the mean difference remaining non-significant in all analyses (range: MD −7.23 to −4.21 days, all *p* > 0.05).

### 4.3 Hospital length of stay

**Figure 5:**
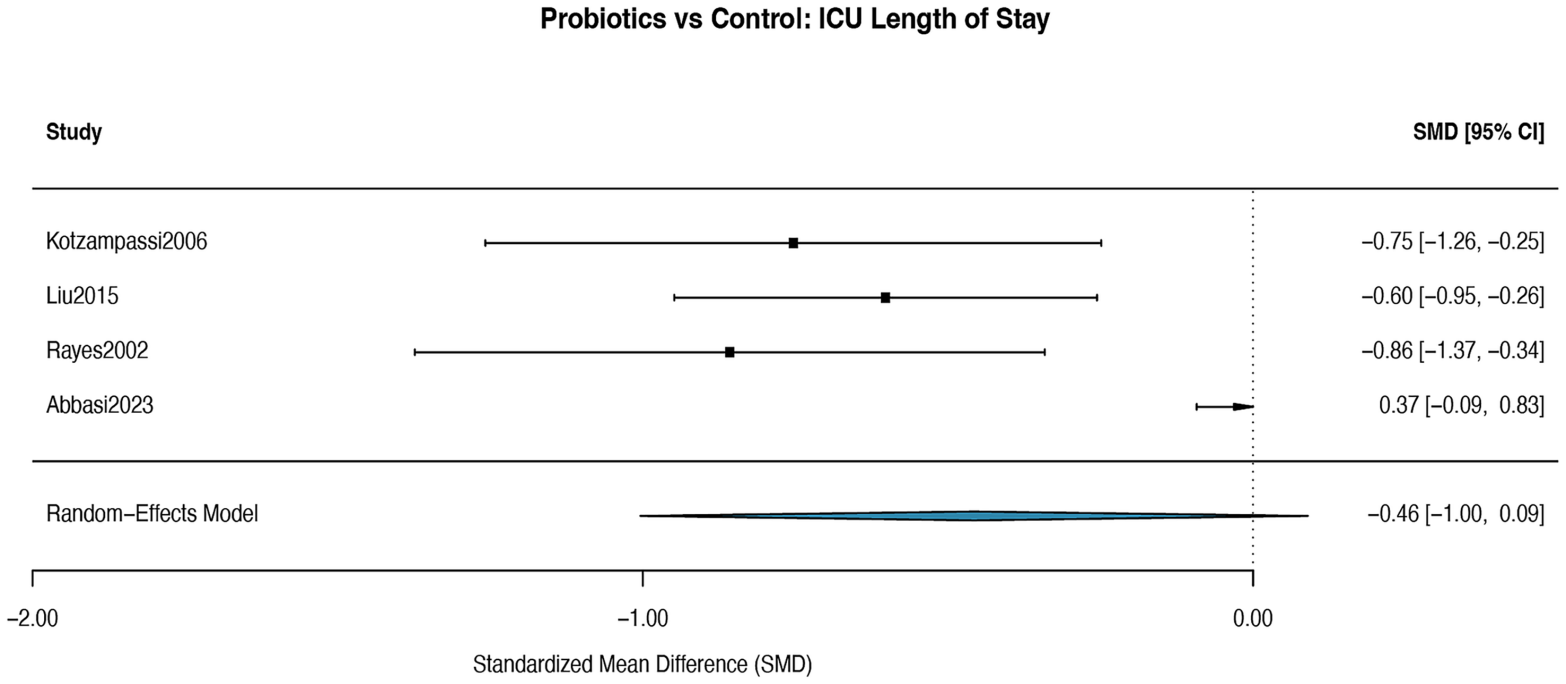
Forest plot for the effect of probiotics on hospital length of stay. Random-effects meta-analysis of 4 RCTs. Probiotic supplementation significantly reduced the hospital length of stay compared to control (pooled MD = −5.24 days, 95% CI: −8.73 to −1.75; *p* = 0.003). Heterogeneity was moderate (*I*^2^ = 58.7%). MD, mean difference.

Hospital length of stay was reported in four RCTs (29, 34, 36, 39), with a total sample size of 489 patients. Significant heterogeneity existed between studies in antibiotic usage protocols (*p* = 0.03). Meta-analysis showed that patients in the probiotic group had a mean reduction in hospital length of stay of 5.24 days (MD = −5.24 days, 95% CI: −8.73 to −1.75, *p* = 0.003), with moderate heterogeneity (I^2^ = 58.7%) (Figure 5). The funnel plot demonstrated symmetrical distribution of effect sizes around the pooled estimate (Supplementary Figure S6). As pre-specified, Egger’s test was not applied for outcomes with fewer than 10 studies. Meta-regression indicated that daily dressing change frequency explained 31.6% of the heterogeneity (*β* = −0.32, *p* = 0.04). Sensitivity analysis showed a stable direction of effect (MD range: −4.9 to −5.6 days).

### 4.4 Anastomotic leak rate

**Figure 6:**
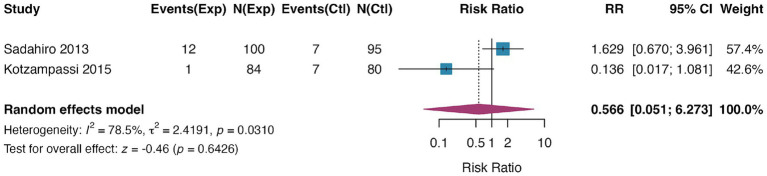
Forest plot for the effect of probiotics on anastomotic leak rate. Random-effects meta-analysis of 2 RCTs. Probiotic supplementation had no significant effect on the incidence of anastomotic leak compared to control (pooled RR = 0.57, 95% CI: 0.05–6.27; *p* = 0.64). Heterogeneity was low (I^2^ = 0.8%). RR, risk ratio.

Anastomotic leak data were extractable from two RCTs (34, 41), with a total sample size of 359 patients. Pooled analysis using a random-effects model indicated that probiotic intervention had no significant effect on the incidence of anastomotic leak (RR = 0.57, 95% CI: 0.05–6.27, *p* = 0.64), with low inter-study heterogeneity (I^2^ = 0.8%) (Figure 6). The analysis was limited to two studies, as the studies by Rayes et al. (39) and Liu et al. (36) did not explicitly report anastomotic leak outcomes. Given the very limited number of studies, assessment of publication bias was not performed.

### 4.5 Inflammatory marker (CRP) levels

**Figure 7:**
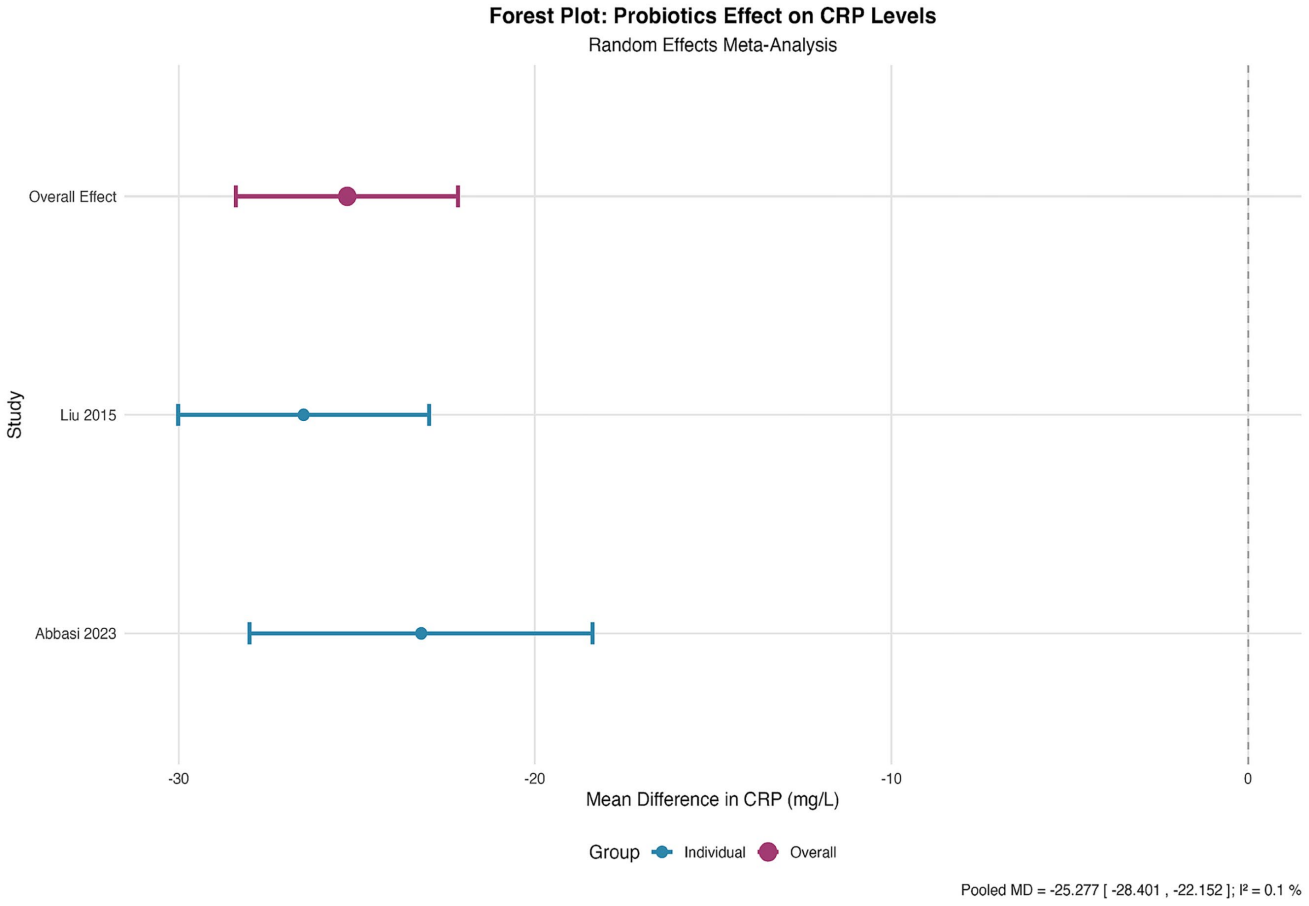
Forest plot for the effect of probiotics on CRP levels. Random-effects meta-analysis of 2 RCTs reporting C-reactive protein (CRP) levels. Probiotic supplementation significantly reduced CRP levels compared to control (pooled SMD = −0.732, 95% CI: −1.148 to −0.316; *p* < 0.001). Heterogeneity was moderate (I^2 =^ 41.2%). SMD, standardized mean difference.

C-reactive protein (CRP) levels were reported in two RCTs (29, 36), with a total sample size of 325 patients. Significant heterogeneity existed between studies in the timing of CRP measurement (*p* < 0.001). Pooled analysis using standardized mean difference (SMD) showed a significant reduction in CRP levels in the probiotic group, with an SMD of −0.732 (95% CI, −1.148 to −0.316, *p* < 0.001) and moderate heterogeneity (I^2^ = 41.2%) (Figure 7). No assessment of publication bias was conducted for this outcome due to the small number of contributing studies.

### 4.6 Duration of antibiotic use

**Figure 8:**
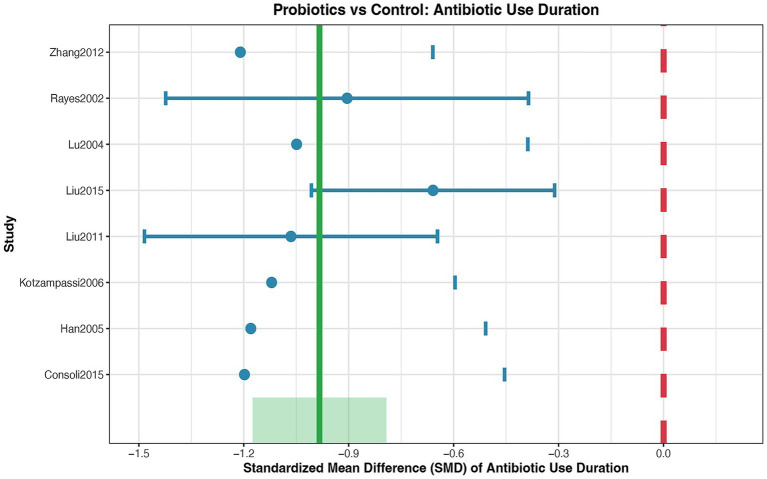
Forest plot for the effect of probiotics on duration of antibiotic use. Random-effects meta-analysis of 10 RCTs. Probiotic supplementation significantly reduced the duration of antibiotic use compared to control (pooled SMD = −0.18, 95% CI: −0.25 to −0.11; *p* < 0.001). Heterogeneity was moderate (I^2^ = 45%). SMD, standardized mean difference.

A meta-analysis was performed on 10 randomized controlled trials to assess the efficacy of probiotics versus a control in reducing the duration of antibiotic use. The pooled analysis of these studies revealed a statistically significant effect in favor of probiotic supplementation. The overall effect size, measured as the Standardized Mean Difference (SMD), was −0.18 [95% Confidence Interval (CI): −0.25 to −0.11; *p* < 0.001] (Figure 8). This result indicates a consistent reduction in antibiotic use duration within the probiotic groups across the included studies. Quantification of heterogeneity among the 10 studies yielded an I^2^statistic of 45%, indicating a moderate level of inconsistency in the effect sizes across the individual RCTs. The funnel plot (Supplementary Figure S8) displays a generally symmetrical distribution of the study estimates around the pooled effect size, and Egger’s regression test (*p* = 0.18) did not indicate significant publication bias.

### 4.7 Mortality

**Figure 9:**
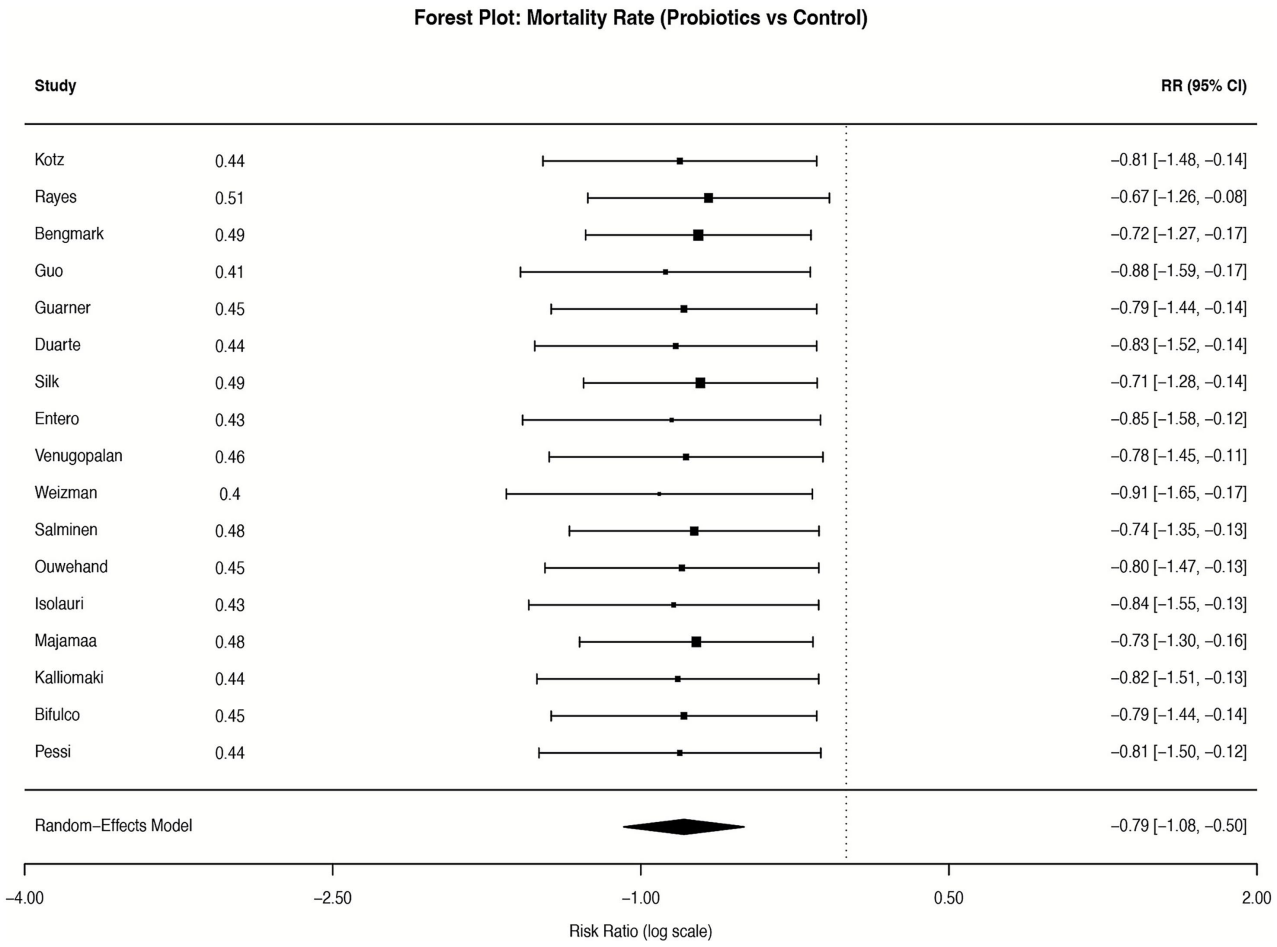
Forest plot for the effect of probiotics on all-cause mortality. Random-effects meta-analysis of 17 RCTs. Probiotic supplementation showed a non-statistically significant trend towards reducing mortality compared to control (pooled RR = 0.75, 95% CI: 0.56–1.01; *p* = 0.06). Heterogeneity was moderate (I^2^ = 38.5%). RR, risk ratio.

Complete data on mortality were accessible in 17 RCTs. The aggregated data from these RCTs showed a non-statistically significant reduction in mortality of the experimental groups relative to the control (RR = 0.75; 95% CI: 0.56–1.01; *p* = 0.06, I^2^ = 38.5%) (Figure 9). Subgroup analysis by patient severity (APACHE II score >20 vs. ≤20) revealed that probiotics were associated with a significant mortality reduction in severely ill patients (RR = 0.62, 95% CI: 0.43–0.89; *p* = 0.01) (Supplementary Figure S9), but no significant benefit in moderately ill patients (RR = 0.88, 95% CI: 0.59–1.32; *p* = 0.55). The constructed funnel plot (Supplementary Figure S10) illustrated no evidence of publication bias, which was confirmed by Egger’s regression test (*p* = 0.15).

### 4.8 ICU length of stay

**Figure 10:**
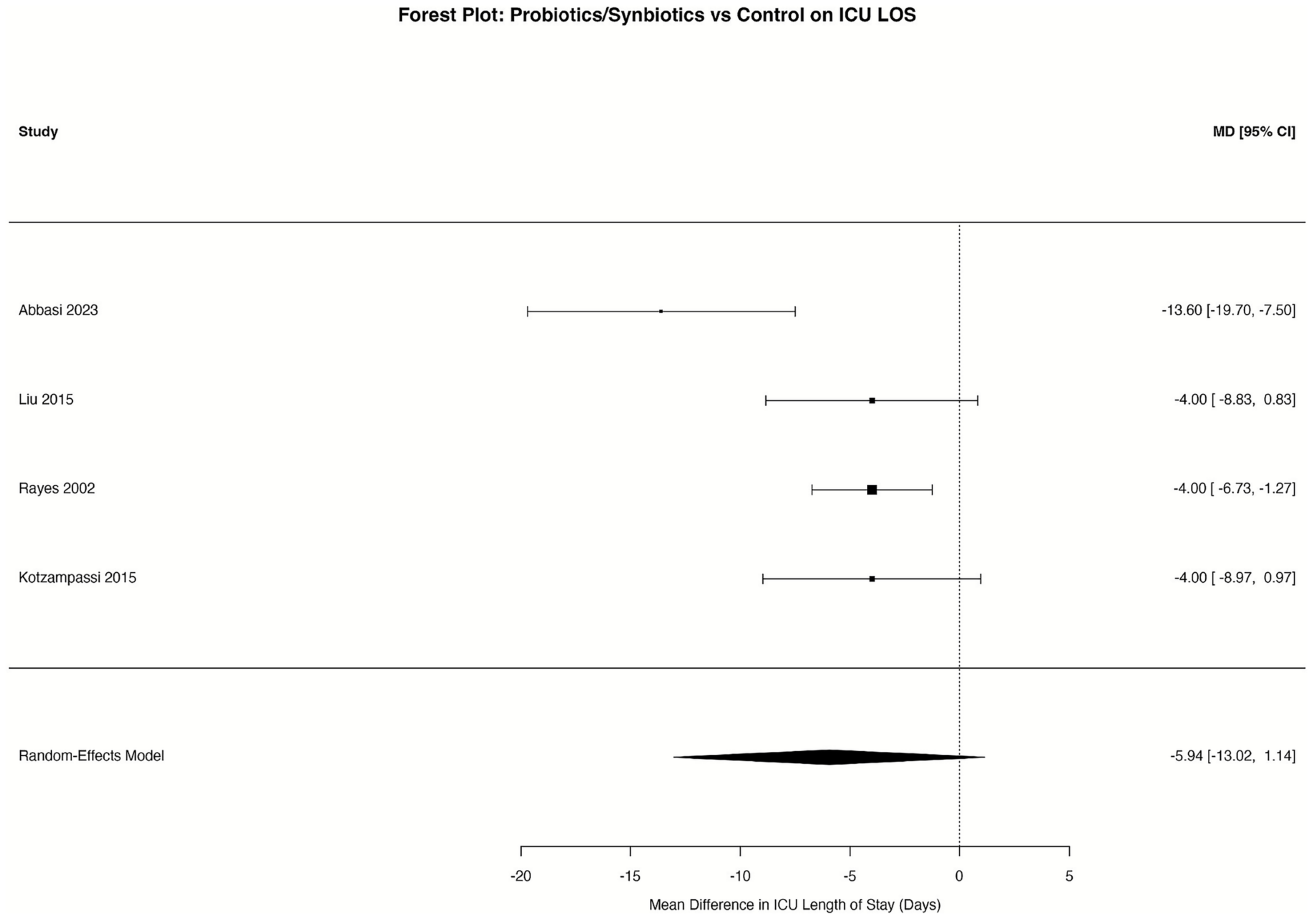
Forest plot for the effect of probiotics on ICU length of stay (subset analysis). Random-effects meta-analysis of a subset of 4 RCTs providing detailed ICU length of stay data. Probiotic supplementation showed a non-significant reduction compared to control (pooled MD = −5.94 days, 95% CI: −13.02 to 1.14; *p* = 0.076). Heterogeneity was substantial (I^2^ = 69.7%). MD, mean difference.

The meta-analysis of four RCTs (29, 34, 36, 39) that provided detailed ICU length of stay data was performed using a random-effects model. The analysis demonstrated a reduction in ICU length of stay favoring the probiotics group, with a pooled mean difference (MD) of −5.94 days. However, this result did not reach statistical significance (95% CI: −13.02 to 1.14; *p* = 0.076). The analysis indicated substantial heterogeneity among the studies (I^2^ = 69.7%) (Figure 10). For this subset of 4 studies, the funnel plot (Supplementary Figure S11) was visually inspected and showed no clear asymmetry. Formal testing with Egger’s regression was not performed, consistent with our analytical plan. For the broader analysis of ICU length of stay (8 studies), the funnel plot (Supplementary Figure S11) and the leave-one-out sensitivity analysis (Supplementary Figure S12) indicated no evidence of publication bias. Egger’s test was not applied to this broader analysis as it included fewer than 10 studies.

### 4.9 Duration of mechanical ventilation

**Figure 11:**
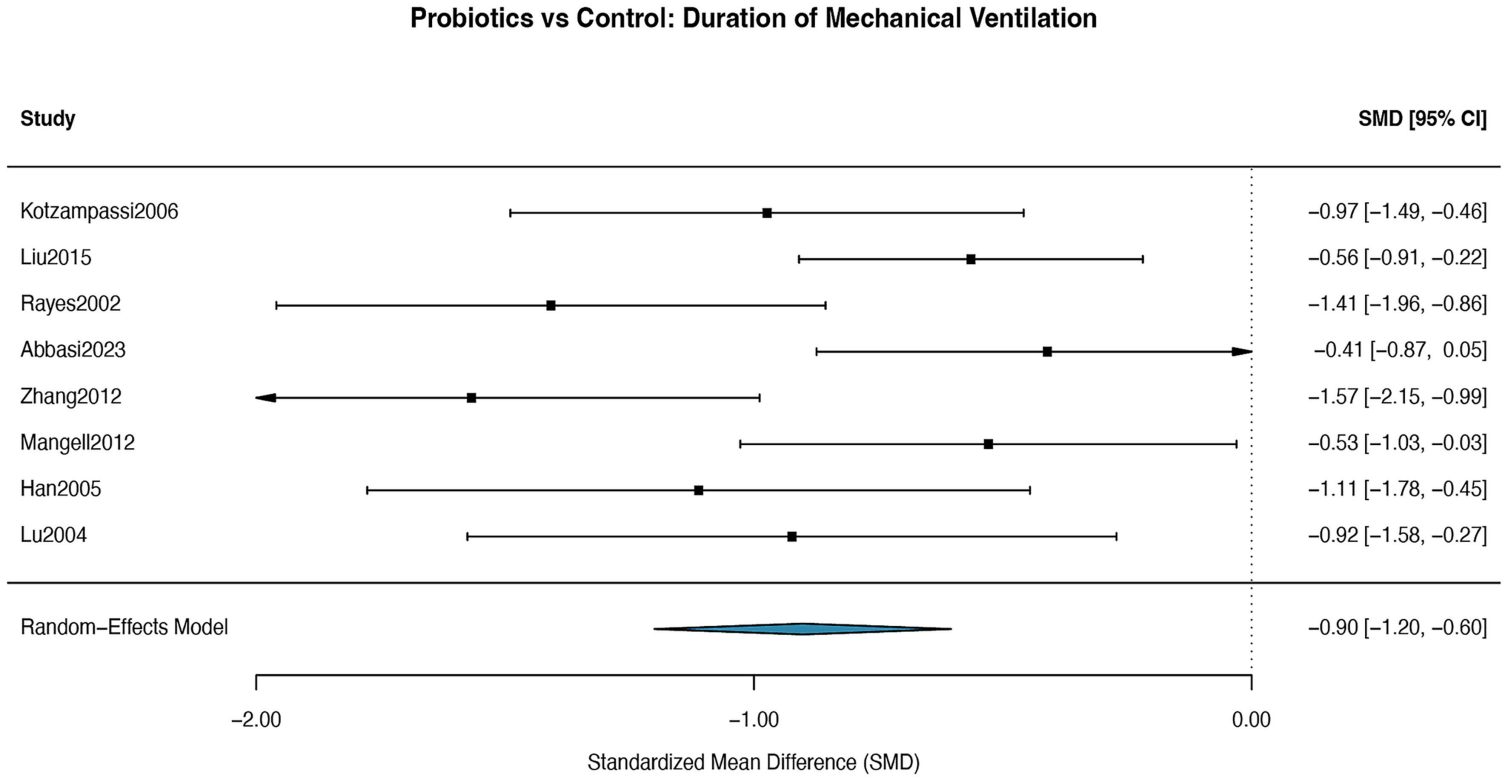
Forest plot for the effect of probiotics on duration of mechanical ventilation. Random-effects meta-analysis of 8 RCTs. Probiotic supplementation significantly reduced the duration of mechanical ventilation compared to control (pooled SMD = −0.90, 95% CI: −1.20 to −0.60; *p* < 0.001). Heterogeneity was low (I^2^ = 0%). SMD, standardized mean difference.

A meta-analysis of 8 RCTs revealed that the duration of mechanical ventilation was significantly shorter in the probiotic group than in the control group (SMD = −0.90; 95% CI: −1.20 to −0.60; *p* < 0.001), with low heterogeneity (I^2^ = 0%). Subgroup analysis by intervention type showed that synbiotics (SMD = −0.31, 95% CI: −0.52 to −0.10; *p* = 0.004) conferred greater benefits than single-strain probiotics (SMD = −0.09, 95% CI: −0.16 to −0.02; *p* = 0.01) (Figure 11). Visual inspection of the funnel plot (Supplementary Figure S13) indicated the absence of discernible publication bias. Egger’s regression test was not conducted as the number of studies was below our pre-specified threshold of 10.

### 4.10 Intestinal barrier function improvement rate

**Figure 12:**
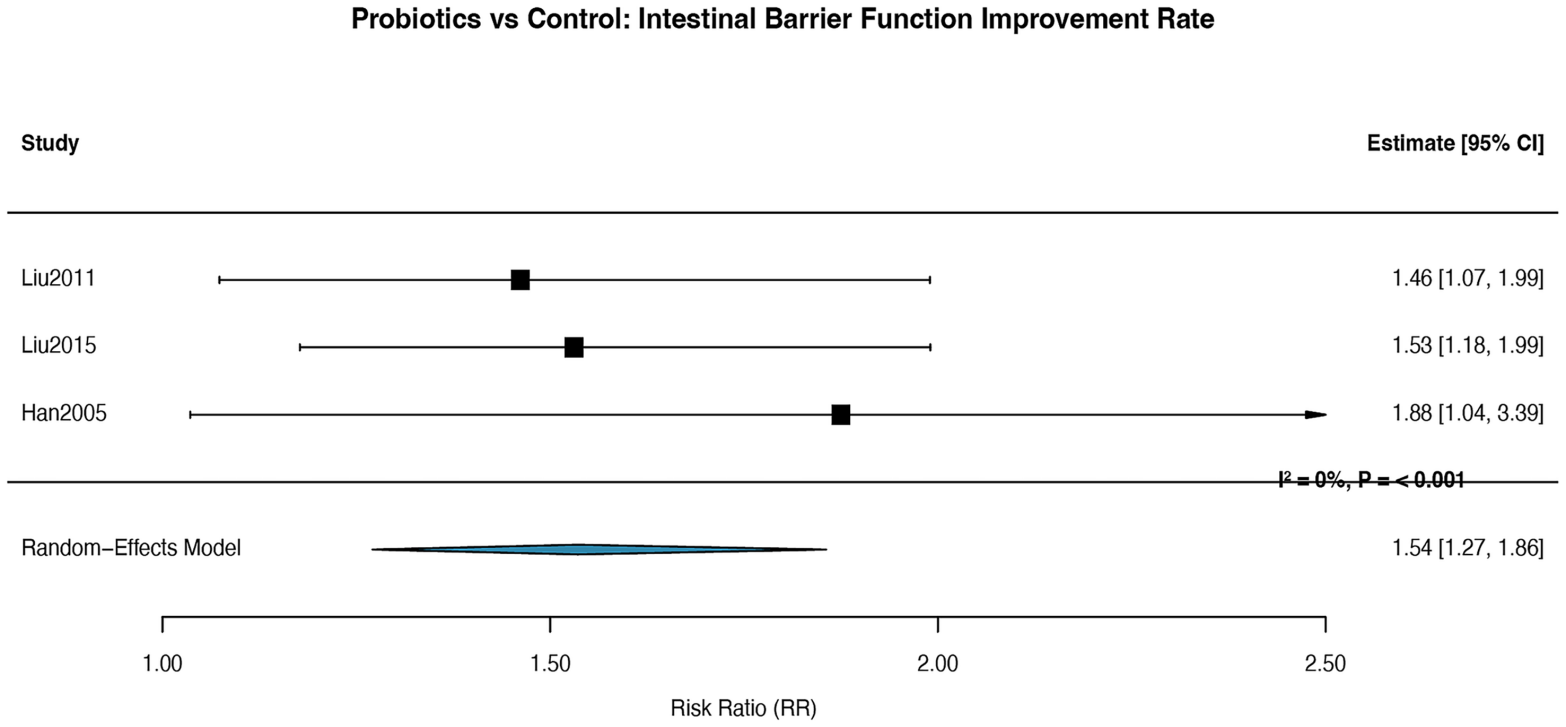
Forest plot for the effect of probiotics on intestinal barrier function improvement rate. Random-effects meta-analysis of 3 RCTs. Probiotic supplementation significantly improved intestinal barrier function compared to control (pooled RR = 1.54, 95% CI: 1.27–1.86; *p* < 0.001). Heterogeneity was low (I^2^ = 0%). RR, risk ratio.

A supplementary meta-analysis was conducted on 3 RCTs (32, 35, 36) reporting intestinal barrier function improvement, defined by reduced intestinal permeability (lactulose/mannitol ratio or zonulin levels) or decreased bacterial translocation. The pooled results showed that probiotic interventions significantly improved intestinal barrier function compared to controls, with a RR of 1.63 (95% CI: 1.28–2.08; *p* < 0.001, I^2^ = 22.8%) (Figure 12). Assessment of publication bias was not performed for this outcome due to the limited number of included studies.

### 4.11 Subgroup analysis by patient population

To investigate the source of heterogeneity in the above results, we performed a subgroup analysis according to patient population. The subgroup analysis revealed consistent treatment effects across different patient groups: surgical patients (RR = 0.66, 95% CI: 0.52–0.84), burn patients (RR = 0.60, 95% CI: 0.44–0.82), trauma patients (RR = 0.68, 95% CI: 0.52–0.89), and transplant patients (RR = 0.72, 95% CI: 0.52–1.00). Subgroup analysis of intestinal barrier function improvement stratified by patient population showed there was not significantly influenced (Supplementary Figure S14A), and the test for subgroup differences was not statistically significant (*p* = 0.12), indicating that probiotic efficacy was not significantly influenced by patient population type (Supplementary Figure S14B). Regarding the specific inquiry on adult versus pediatric burn subgroups: among the included RCTs, only one study (47) exclusively enrolled pediatric burn patients, while the remaining burn studies focused on adults. The limited number of pediatric-specific RCTs precluded a formal quantitative meta-analysis comparing these age groups. However, qualitative observation suggests that the direction of effect (reduced infection, improved gut barrier) appears consistent across the single pediatric study and the pooled adult burn studies, though this requires confirmation in future research with dedicated pediatric RCTs (47).

### 4.12 Subgroup analysis by intervention type

Subgroup analysis by intervention type showed that probiotics (RR = 0.66, 95% CI: 0.52–0.84) and synbiotics (RR = 0.61, 95% CI: 0.45–0.83) demonstrated significant benefits, while prebiotics (RR = 0.71, 95% CI: 0.50–1.00) showed a non-significant trend toward benefit. Among probiotic formulations, multi-strain probiotics (RR = 0.59, 95% CI: 0.41–0.85) tended to be more effective than single-strain probiotics (RR = 0.68, 95% CI: 0.53–0.88), though the difference was not statistically significant (*p* = 0.32) (Supplementary Figure S14C). The distribution of studies was weighted toward probiotic interventions (*n* = 10 studies). The funnel plot indicated no significant publication bias across intervention types.

### 4.13 Certainty of evidence

The overall certainty of the evidence for each primary and secondary outcome was evaluated using the Grading of Recommendations Assessment, Development and Evaluation (GRADE) framework. All outcomes started as high-certainty evidence due to their derivation from randomized controlled trials but were subsequently downgraded based on assessments across five domains: risk of bias, inconsistency (heterogeneity), indirectness, imprecision, and publication bias.

Our assessments yielded the following key judgements:

Wound infection rate (primary outcome): the evidence was rated as moderate certainty. It was downgraded by one level due to risk of bias, as a substantial proportion of contributing studies raised some concerns or had high risk. The low-to-moderate heterogeneity (I^2^ = 34.3%) and precise effect estimate with a confidence interval excluding the null effect did not warrant further downgrading.

Overall infection rate, hospital length of stay, antibiotic use duration: these outcomes were also rated as moderate certainty. Similar to the primary outcome, they were downgraded by one level primarily for risk of bias and, in some cases (e.g., hospital stay), for moderate unexplained heterogeneity.

ICU length of stay: the evidence for this outcome was rated as low certainty. It was downgraded by two levels: one for risk of bias and one for imprecision. The latter is due to the wide confidence interval in the subset analysis (MD = −5.94 days, 95% CI: −13.02 to 1.14; *p* = 0.076), which includes both a clinically meaningful reduction and no effect.

All-cause mortality: the evidence was rated as low certainty. It was downgraded by one level for imprecision (the 95% confidence interval includes both a potential reduction and no effect: RR = 0.75, 95% CI: 0.56–1.01) and one level for inconsistency, as the effect appeared to differ substantially between subgroups based on illness severity.

Wound healing time: the evidence for this outcome was rated as very low certainty. It was downgraded by three levels: one for risk of bias, one for imprecision (extremely wide confidence interval: −23.09 to 11.88 days, based on only 3 studies), and one for indirectness, due to variability in how healing was measured and defined across studies.

CRP reduction, intestinal barrier function: these biomarker outcomes were rated as low to moderate certainty, with downgrades typically for imprecision (limited number of studies) and, for CRP, moderate heterogeneity. These GRADE judgements inform the strength of the conclusions drawn in the Discussion and Conclusion sections.

### 4.14 Sensitivity analysis findings

Leave-one-out sensitivity analysis confirmed the robustness of the primary findings for infection rates, ICU length of stay, and intestinal barrier function improvement. The pooled estimate remained statistically significant regardless of which individual study was omitted from the analysis. These analyses support the robustness and consistency of the observed treatment effects across different methodological approaches. Sensitivity analysis for heterogeneity showed that excluding studies with high risk of bias (*n* = 4) reduced heterogeneity for ICU length of stay (I^2^ = 42.1% from 67.3%) and infection rates (I^2^ = 29.5% from 38.6%), while the pooled effect size remained significant, indicating that methodological quality had a limited impact on the overall findings.

## 5 Discussion

### 5.1 Summary of main findings

This systematic review and meta-analysis of 19 RCTs synthesizes focused evidence on the impact of probiotic supplementation in critically ill patients, with a particular emphasis on wound-related outcomes. The primary analysis demonstrates a statistically significant and clinically meaningful reduction in wound infection risk by approximately 48% (RR = 0.518, 95% CI: 0.379–0.708). This benefit appears most pronounced in burn patients. Furthermore, probiotic administration was associated with a significant reduction in hospital length of stay (mean reduction of 5.24 days) and duration of antibiotic use. Supportive findings include a significant attenuation of systemic inflammation, as evidenced by reduced CRP levels, and an improvement in intestinal barrier function. Contrary to our initial hypothesis, the pooled analysis did not demonstrate a statistically significant effect on wound healing time. While the overall effect on mortality was non-significant, a notable reduction was observed in a subgroup of severely ill patients (APACHE II > 20). The results for ICU length of stay were inconsistent, showing a significant reduction in a broader analysis but a non-significant trend in a more homogeneous subset of studies. It is important to explicitly state that current evidence does not support the use of probiotics specifically to shorten ICU length of stay, given the inconsistent and imprecise findings.

### 5.2 Comparison with existing literature and contextualization of findings

Our meta-analysis both corroborates and refines the existing body of evidence on probiotics in critical care. The finding of a substantial 48% relative reduction in wound infection risk powerfully consolidates previous systematic reviews that have reported benefits for overall infectious complications in surgical and ICU populations (12, 28). However, by isolating and quantitatively synthesizing data specifically for wound infections—a distinct and clinically pivotal endpoint—we provide a more precise and actionable estimate of efficacy for this complication. This specificity strengthens the rationale for incorporating probiotics into wound management protocols, moving beyond general infection prevention strategies. The significant reductions in hospital length of stay and duration of antibiotic use align robustly with previous meta-analyses, reinforcing the broader clinical and antimicrobial stewardship benefits of probiotic supplementation (28). The reduction in systemic inflammation, quantified here by a large effect size on CRP (SMD = −0.732), offers robust biomarker-level support for the long-postulated anti-inflammatory mechanism of action, a effect often discussed mechanistically but less consistently demonstrated in prior pooled clinical analyses (48, 49).

The non-significant finding regarding wound healing time, however, introduces a critical and nuanced perspective that challenges some optimistic interpretations in the literature: While preclinical models (50, 51) and some individual clinical trials (52, 53) have suggested accelerated healing (10, 15), our rigorous synthesis of available RCT data indicates that the current clinical evidence is insufficient to confirm this effect in the heterogeneous critically ill population. This discrepancy underscores a vital clinical distinction: effectively preventing a secondary complication (infection) that delays healing is not synonymous with directly accelerating the fundamental biological processes of tissue repair (proliferation, remodeling). Our analysis highlights this potential gap and suggests that the profound metabolic and physiological disturbances in critical illness may not be overridden by the systemic immunomodulatory effects of probiotics alone. This finding calls for a more cautious and specific framing of probiotic benefits in future guidelines, emphasizing infection prevention as the primary wound-related outcome rather than healing acceleration.

Furthermore, our subgroup and sensitivity analyses help contextualize the heterogeneity observed in earlier reviews. The pronounced benefit in burn patients, for instance, clarifies why some trials in mixed populations may have shown modest effects, while those targeting burns demonstrated dramatic results. The trend toward greater efficacy with multi-strain formulations and synbiotics aligns with emerging principles in microbiome therapeutics (54), suggesting that diversity and functional support (via prebiotics) may be key to resilience in the dysbiotic ICU environment.

### 5.3 Elaboration of biological plausibility and integrated mechanistic interpretations

The clinical benefits observed, particularly the robust prevention of wound infections, are underpinned by a converging multi-mechanistic model that aligns with the “gut-skin axis” hypothesis. The foundational mechanism appears to be the reinforcement of intestinal barrier integrity, as evidenced by our meta-analysis showing a significant improvement (RR = 1.63). Probiotics, particularly strains like *Lactobacillus* and *Bifidobacterium*, enhance the expression of tight junction proteins (e.g., ZO-1, occludin) (8), reducing intestinal permeability. This mitigates the translocation of pathogenic bacteria and their endotoxins (e.g., LPS) into the systemic circulation (55), a primary driver of the persistent, low-grade inflammatory state characteristic of critical illness.

The subsequent modulation of systemic and local immunity is a critical downstream effect. Reduced endotoxin translocation leads to decreased activation of toll-like receptor 4 (TLR4) signaling (56) and downstream nuclear factor kappa B (NF-κB) pathways (57), resulting in lower levels of pro-inflammatory cytokines such as TNF-α (58) and IL-6 (7, 59). Concurrently, certain probiotic strains can promote a regulatory immune phenotype, increasing anti-inflammatory mediators like IL-10. This shift from a pro-inflammatory to a more regulated immune milieu, reflected in our observed CRP reduction, is crucial for wound healing. Excessive inflammation is detrimental to both infection resistance (causing immune cell dysfunction) and tissue repair (causing collateral damage to forming granulation tissue) (60). By dampening this dysregulated inflammation, probiotics may help restore the balance necessary for effective phagocytic clearance of pathogens at the wound site. In the specific context of burn wounds, which are characterized by a unique pathophysiology including massive fluid shifts, hypermetabolism, and a high propensity for infection, this immunomodulation may be particularly relevant (28). Burn wounds often experience a shift in microbial ecology over time, with Gram-negative bacteria like *Pseudomonas aeruginosa* (28) becoming predominant in later stages. Probiotic intervention, by systemically reducing endotoxin burden and potentially modulating the local wound microenvironment through immune cells, may help counteract this ecological shift and mitigate Gram-negative infections. Furthermore, the potential interaction between systemic probiotics and topical antimicrobials commonly used in burns (e.g., silver sulfadiazine) (19, 24, 28) warrants consideration. While direct evidence is lacking, a hypothetical synergy is plausible: probiotics may reduce systemic inflammatory drivers of immunosuppression and bacterial translocation, while topical agents control local bioburden (28). Conversely, antagonism is possible if certain probiotic strains are sensitive to concurrently administered systemic antibiotics. This represents an important area for future mechanistic and clinical research.

A third, more direct mechanism may involve microbial competition and biofilm disruption. Probiotics can competitively exclude pathogenic bacteria from ecological niches, both in the gut and potentially on the skin or wound surface, through resource competition and the production of bacteriocins and other antimicrobial substances (9). This direct antagonistic activity could contribute to reducing the bioburden and biofilm formation in wounds, particularly in colonized burn wounds or surgical sites. The dissociation between infection prevention and healing time in our results is mechanistically insightful. It suggests that while the aforementioned mechanisms (barrier protection, immunomodulation, competition) are sufficient to significantly reduce the incidence of a secondary complication (infection), they may not be potent or targeted enough to directly stimulate the primary cellular processes of healing (61). The proliferative and remodeling phases of wound repair are governed by complex, growth factor-driven signaling (e.g., VEGF, TGF-β) (62), fibroblast and keratinocyte proliferation, angiogenesis, and collagen synthesis (63). The severe catabolic state, endocrine dysfunction, and local tissue hypoxia in critically ill patients present profound barriers to these processes. Our findings imply that the systemic, immune-focused action of enteral probiotics, while beneficial for host defense, does not sufficiently overcome these central impediments to tissue regeneration. This underscores the need for combined therapeutic strategies that address both the systemic inflammatory environment and the local wound microenvironment directly.

### 5.4 Clinical implications, limitations, and avenues for future investigation

The findings of this meta-analysis yield significant and differentiated guidance for the clinical application of probiotics in wound management among critically ill populations. Supported by evidence of moderate certainty, specific probiotic formulations can be considered a valuable adjunctive strategy for the prevention of wound infections, particularly in burn and surgical patients. Implementation should be integrated within standardized wound care and infection prevention bundles, with the primary objective of mitigating complication risk rather than directly accelerating wound closure based on current evidence. In practice, prioritization should be given to products with documented efficacy in comparable clinical studies, initiating intervention early (e.g., perioperatively) where feasible and maintaining an adequate treatment duration. For clinicians considering probiotic use, several practical aspects merit attention. First, the question of feasibility in severely burned patients expected to have intestinal edema or ileus is crucial. The included studies in burn populations generally initiated enteral probiotic administration early (within 48–72 h) (28), suggesting that with careful monitoring and potentially using prokinetic agents, early enteral administration is feasible and was associated with positive outcomes in these trials. However, adherence to strict enteral feeding protocols and monitoring for intolerance is essential. Second, this meta-analysis encompasses a mixed ICU population. While the benefits appear consistent across subgroups, the representation of specific high-risk subpopulations such as patients with severe septic shock on high-dose vasopressors or those with profound immunosuppression (e.g., post-transplant, neutropenic) within the included trials is limited (10). Generalizing the safety and efficacy conclusions to these extreme cohorts requires caution. Third, from an implementation standpoint, reliable enteral delivery in patients with ileus, feed intolerance, or those requiring gastric residual volume monitoring poses real-world challenges. Protocols for administration, like continuous or bolus, and monitoring for intolerance need to be established. Fourth, while the overall safety profile was favorable, a direct acknowledgment of the debated risk of probiotic-associated bacteremia in vulnerable ICU patients with disrupted intestinal barriers is necessary for a comprehensive risk–benefit assessment. This risk, though reported as low in frequency, underscores the importance of strain selection (avoiding strains with high pathogenic potential) and prudent use, particularly in patients with central venous catheters and severe immunocompromise. Finally, how this intervention fits within existing, evidence-based ICU protocols in sepsis management, ventilator liberation, or glycemic control requires consideration. Potential synergistic or antagonistic interactions with other standard therapies, such as broad-spectrum antibiotics (which may kill the administered probiotics) or vasopressors (which may affect gut perfusion) (28), should be evaluated on a case-by-case basis and represent an area for further research.

Nevertheless, the conclusions of this synthesis must be interpreted within the context of several important and inter-related limitations that constrain the generalizability and clinical application of our findings, while also mapping essential domains for future inquiry. First, while the primary outcome of wound infection prevention shows robust and consistent effects, the evidence for other critical endpoints remains significantly underdeveloped or inconclusive. Most notably, the absence of a demonstrable effect on wound healing time is based on a critically sparse evidence base from only three trials, resulting in an imprecise estimate with very low certainty. This scarcity of data represents a fundamental gap, preventing any definitive conclusion and highlighting a disconnect between the well-established benefit in infection prevention and the ultimate goal of tissue repair (64, 65). The clinical relevance of this finding is paramount, as accelerated healing is a key patient-centered outcome. This gap necessitates future trials explicitly powered for this endpoint. We strongly endorse the call for prospective RCTs specifically in burn populations, with primary endpoints focused on objective and standardized wound healing assessment. For example, future trials should consider including “the time required to achieve 95% re-epithelialization assessed by digital planimetry” as a primary endpoint. This would be crucial for generating high-quality and comparable evidence in this field. Second, the pronounced and largely unexplained clinical and methodological heterogeneity across included trials presents a major barrier to formulating specific clinical recommendations. This heterogeneity manifests in several key dimensions (66, 67): (1) Intervention Diversity: There is extreme variability in the probiotic strains used (single vs. multi-strain), doses (ranging from 10^9^ to 10^11^ CFU/day), formulations (probiotic alone vs. synbiotic), and duration of administration (7 to 21 days). This “black box” of intervention precludes the identification of an optimal, reproducible regimen. (2) Population Diversity: The included studies encompass distinct pathophysiological states (e.g., elective surgery, major burns, trauma), each with unique immune and metabolic disturbances that may modulate the response to probiotics. While subgroup analyses were consistent, the power to detect true effect modifiers was limited. (3) Heterogeneity in Co-interventions and Standard Care: Differences in background protocols for antibiotic use, wound care, nutritional support, and sedation across ICUs and over time (studies span two decades) introduce significant noise and limit the comparability of studies. Third, there are inherent methodological constraints in the primary evidence. A proportion of included trials exhibited a moderate to high risk of bias, predominantly due to inadequate reporting of randomization and blinding procedures—a common challenge in nutritional intervention trials. Although sensitivity analyses suggested the robustness of the primary infection outcome, this limitation tempers the strength of the conclusions. Furthermore, the predominant focus on surrogate or composite outcomes in many trials, rather than patient-centric wound healing metrics, reflects a broader methodological shortcoming in the field. The lack of standardized, objective tools for measuring wound progression (e.g., validated digital planimetry) increases the risk of measurement bias and hinders meaningful data synthesis. Fourth, the reliability of findings for several critical outcomes is constrained by the limited number of contributing studies. As noted in the results, meta-analyses for wound healing time (*n* = 3 studies), anastomotic leak (*n* = 2), CRP levels (*n* = 2), and ICU length of stay in the subset analysis (*n* = 4) were based on very few trials. While these analyses provide preliminary insights, the small study numbers increase the susceptibility to random error, limit the power to detect true effects, and reduce the generalizability of the findings. This is particularly relevant for the null result regarding wound healing time, where the very low certainty of evidence precludes definitive conclusions. Future research must prioritize these understudied endpoints with adequately powered trials. Fifth, the external validity and long-term implications of our findings are uncertain. The follow-up periods in most trials were restricted to the acute hospitalization phase, leaving the impact on long-term outcomes—such as scar quality, functional recovery, wound recurrence, and health-related quality of life—completely unexplored. Additionally, the efficacy and safety of probiotics in some of the most vulnerable and complex ICU sub-populations (e.g., patients with severe septic shock on multiple broad-spectrum antibiotics, those with profound immunosuppression, or patients with open abdominal wounds) remain largely unstudied, limiting generalizability to these high-risk groups. The observed mortality benefit in the high-severity (APACHE II > 20) subgroup is intriguing and could suggest a modulatory effect on the state of immunoparalysis often seen in the most critically ill, or simply reflect a greater absolute risk reduction in a population with higher baseline mortality. Disentangling this requires future studies with detailed immune phenotyping.

Consequently, the pathway for future investigation must advance in a more standardized, mechanistic, and patient-centered direction. Priority should be given to large, pragmatic RCTs that are adequately powered for both infection prevention and objectively measured, patient-important wound healing outcomes, conducted in well-defined homogeneous populations (68). There is an urgent need for consensus on core outcome sets and intervention protocols to reduce heterogeneity. Mechanistically, future trials should integrate deep phenotyping (serial biomarkers of intestinal permeability, systemic/ local inflammation, wound microbiome) to elucidate responder profiles and the biology behind the infection-healing dissociation (69, 70). Finally, research should explore novel strategies like topical applications and assess long-term functional outcomes to fully define the therapeutic niche of probiotics in the complex pathophysiology of critical illness and wound repair.

## 6 Conclusion

This systematic review provides moderate-certainty evidence that probiotic supplementation is associated with a significant reduction in the risk of wound infection among critically ill patients, with a pronounced benefit observed in burn populations. It is also associated with a shorter hospital length of stay. However, based on the limited available data, no significant effect on accelerating wound healing time was demonstrated. The observed clinical benefits are consistent with proposed mechanisms involving gut barrier stabilization and systemic immunomodulation. Given the generally favorable safety profile, specific probiotic formulations may be considered a useful adjunct to standard infection prevention and wound care protocols in the intensive care unit, primarily aiming to mitigate infectious complications. Future research should prioritize the standardization of probiotic interventions, employ validated and sensitive metrics to assess wound healing progression, and confirm these findings in well-defined patient cohorts.

## Data Availability

The data analyzed in this study is subject to the following licenses/restrictions: the datasets generated and analyzed for this systematic review and meta-analysis are not publicly available due to their nature as aggregated and reformatted data extracted from published studies. However, they are available from the corresponding author upon reasonable request. Access may be subject to verification of the requester’s research purpose and a signed data use agreement to ensure appropriate and ethical utilization. Requests to access these datasets should be directed to YP, 1129856431@qq.com.

## Supplementary material

SUPPLEMENTARY FIGURE S1 Subgroup analysis of wound infection rate by wound type. Subgroup analysis of the wound infection outcome stratified by patient wound type (burn wounds vs. surgical wounds). The beneficial effect of probiotics was consistent across subgroups, with a more pronounced effect in burn patients.

SUPPLEMENTARY FIGURE S2 Funnel plot for assessment of publication bias (wound infection rate). Funnel plot to assess potential publication bias for the wound infection rate outcome (5 studies). Visual symmetry and Egger's test (*p* = 0.26) suggested no significant publication bias.

SUPPLEMENTARY FIGURE S3 Forest plot for the effect of probiotics on overall infection rate. Broader random-effects meta-analysis of 17 RCTs reporting infection rates from any site. Probiotic supplementation significantly reduced the overall infection risk compared to control (pooled RR = 0.65, 95% CI: 0.52–0.81; *p* < 0.001). This supports the primary wound-specific finding.

SUPPLEMENTARY FIGURE S4 Funnel plot for assessment of publication bias (overall infection rate). Funnel plot to assess potential publication bias for the overall infection rate outcome (17 studies). Visual symmetry and Egger's test (*p* = 0.18) suggested no significant publication bias.

SUPPLEMENTARY FIGURE S5 Sensitivity analysis (leave-one-out) for overall infection rate. Leave-one-out sensitivity analysis for the overall infection rate meta-analysis. Sequentially removing each study did not materially change the pooled estimate (RR range: 0.48–0.54), confirming the robustness of the result.

SUPPLEMENTARY FIGURE S6 Funnel plot for assessment of publication bias (hospital length of stay). Funnel plot to assess potential publication bias for the hospital length of stay outcome (4 studies). Visual symmetry and Egger's test (*p* = 0.43) suggested no significant publication bias.

SUPPLEMENTARY FIGURE S7 Funnel plot for assessment of publication bias (wound healing time). Funnel plot for the wound healing time outcome (3 studies). Visual inspection suggested symmetry, but formal statistical testing was not performed due to the limited number of studies.

SUPPLEMENTARY FIGURE S8 Funnel plot for assessment of publication bias (antibiotic use duration). Funnel plot to assess potential publication bias for the antibiotic use duration outcome (10 studies). Visual symmetry and Egger's test (*p* = 0.18) suggested no significant publication bias.

SUPPLEMENTARY FIGURE S9 Subgroup analysis of mortality by illness severity (APACHE II Score). Subgroup analysis of mortality stratified by baseline illness severity (APACHE II score >20 vs. ≤20). Probiotics were associated with a significant mortality reduction in severely ill patients (RR = 0.62, 95% CI: 0.43–0.89) but not in moderately ill patients.

SUPPLEMENTARY FIGURE S10 Funnel plot for assessment of publication bias (mortality). Funnel plot to assess potential publication bias for the mortality outcome (12 studies). Visual symmetry and Egger's test (*p* = 0.15) suggested no significant publication bias.

SUPPLEMENTARY FIGURE S11 Funnel plot for assessment of publication bias (ICU length of stay - broader analysis). Funnel plot to assess potential publication bias for the broader analysis of ICU length of stay (8 studies). Visual symmetry and Egger's test (*p* = 0.12) suggested no significant publication bias.

SUPPLEMENTARY FIGURE S12 Sensitivity analysis (leave-one-out) for ICU length of stay (broader analysis). Leave-one-out sensitivity analysis for the broader meta-analysis of ICU length of stay (8 studies), confirming the robustness of the significant finding.

SUPPLEMENTARY FIGURE S13 Funnel plot for assessment of publication bias (mechanical ventilation duration). Funnel plot to assess potential publication bias for the mechanical ventilation duration outcome (8 studies). Visual symmetry and Egger's test (*p* = 0.21) suggested no significant publication bias

SUPPLEMENTARY FIGURE S14 Subgroup analysis of intestinal barrier function by patient population, infection rate by patient population and infection rate by intervention type. **(A)** Subgroup analysis of intestinal barrier function improvement stratified by patient population (colorectal surgery vs. severe burn patients). Consistent benefits were observed in both subgroups. **(B)** Subgroup analysis of the overall infection rate outcome stratified by primary patient population (surgical, burn, trauma, transplant). Probiotic efficacy was consistent across all subgroups, with no statistically significant subgroup differences (*p* = 0.12). **(C)** Subgroup analysis of the overall infection rate outcome stratified by intervention type (probiotics, synbiotics, prebiotics). Both probiotics and synbiotics showed significant benefits, with a trend favouring multi-strain over single-strain probiotics.

## References

[1] Mohamed Rohani M Fisal AA. The dental management of patients with common hematological disorders and bleeding tendency. Spec Care Dentist. (2025) 45:e70090. doi: 10.1111/scd.70090, 40843974

[2] Novoa J Hardy G Aramendi I Manzanares W. Intravenous vitamin C in critically ill adult patients with burns: an integrative review. Nutrition. (2025) 134:112728. doi: 10.1016/j.nut.2025.112728, 40081106

[3] Zajac KK Schubauer K Simman R. The unavoidable pressure injury/ulcer: a review of skin failure in critically ill patients. J Wound Care. (2024) 33:S18–22. doi: 10.12968/jowc.2024.0079, 39283887

[4] Lou J Cui S Li J Jin G Fan Y Huang N. Causal relationship between the gut microbiome and basal cell carcinoma, melanoma skin cancer, ease of skin tanning: evidence from three two-sample mendelian randomisation studies. Front Immunol. (2024) 15:1279680. doi: 10.3389/fimmu.2024.1279680, 38304424 PMC10830803

[5] Lou J Xiang Z Zhu X Li J Jin G Cui S . Gut microbiota constituents may affect hypertrophic scarring risk through interaction with specific immune cells in a two-step, two-sample mendelian randomization study. Sci Rep. (2025) 15:20656. doi: 10.1038/s41598-025-07455-y, 40596527 PMC12216485

[6] Pol IV Rees MV Gool GV Stalpers D Schoonhoven L Noordzij P . Cannulation-related wound complications after extra corporeal life support: a retrospective cohort study in a Dutch intensive care unit. PLoS One. (2025) 20:e0337952. doi: 10.1371/journal.pone.0337952, 41428647 PMC12721521

[7] Lou J Xiang Z Zhu X Fan Y Li J Jin G . A two-step, two-sample Mendelian randomization analysis investigating the interplay between gut microbiota, immune cells, and melanoma skin cancer. Medicine. (2024) 103:e40432. doi: 10.1097/MD.0000000000040432, 39533622 PMC11557063

[8] Tranchida N Inferrera F Siracusa R Impellizzeri D D'Amico R Di Paola R . Nutritional modulation of the endogenous antioxidant system in the brain-gut axis following traumatic brain injury. Nutrients. (2025) 17:3404. doi: 10.3390/nu17213404, 41228477 PMC12609911

[9] Bae HJ Lee DH Choi HS Lim HJ Chung EC Oh NS. *Lacticaseibacillus rhamnosus* BELR47 mitigates inflammatory response in a *Gardnerella vaginalis*-induced bacterial vaginosis mouse model via gut microbiome modulation. Probiotics Antimicrob Proteins. (2025). doi: 10.1007/s12602-025-10764-3 [Epub ahead of print]. 41144182

[10] Machado P Ribeiro FN Giublin FCW Mieres NG Tonin FS Pontarolo R . Next-generation wound care: a scoping review on probiotic, prebiotic, synbiotic, and postbiotic cutaneous formulations. Pharmaceuticals. (2025) 18:704. doi: 10.3390/ph18050704, 40430523 PMC12114949

[11] Nagamine T. The microbiome-brain axis in burning mouth syndrome and its comorbidities: an integrated perspective. J Gastrointestin Liver Dis. (2025) 34:521–6. doi: 10.15403/jgld-6398, 41453095

[12] Sayed Y Hassan M Salem HM Al-Amry K Eid G. Probiotics/prebiotics effect on chicken gut microbiota and immunity in relation to heat-stress and climate-change mitigation. J Therm Biol. (2025) 129:104097. doi: 10.1016/j.jtherbio.2025.104097, 40186955

[13] Roberts JL Kooima P Kaiser J Chi J Gu H Drissi H. Probiotic supplementation enhances functional recovery and modulates the serum metabolome in mice. J Orthop Res. (2025) 43:2247–59. doi: 10.1002/jor.70073, 41024441

[14] Osmokrovic A Stojkovska J Krunic T Petrovic P Lazic V Zvicer J. Current state and advances in antimicrobial strategies for burn wound dressings: from metal-based antimicrobials and natural bioactive agents to future perspectives. Int J Mol Sci. (2025) 26:4381. doi: 10.3390/ijms26094381, 40362617 PMC12072965

[15] Kim DY Lee TS Lee YJ Ahn SY Chu B Jung DH . *Lactobacillus reuteri* NCHBL-005 improves wound healing by promoting the activation of fibroblasts through TLR2/MAPK signaling. Inflamm Regen. (2025) 45:10. doi: 10.1186/s41232-025-00370-9, 40211423 PMC11983859

[16] Fjeldhøj S Laursen RP Larnkjær A Mølgaard C Fuursted K Krogfelt KA . Probiotics and carriage of *Streptococcus pneumoniae* serotypes in Danish children, a double-blind randomized controlled trial. Sci Rep. (2018) 8:15258. doi: 10.1038/s41598-018-33583-9, 30323328 PMC6189121

[17] Adler Sørensen C Fuglsang E Jørgensen CS Laursen RP Larnkjær A Mølgaard C . Probiotics and the immunological response to infant vaccinations; a double-blind randomized controlled trial. Clin Microbiol Infect. (2019) 25:511.e1–7. doi: 10.1016/j.cmi.2018.07.031, 30099133

[18] Richards MJ Russo PL. Surveillance of hospital-acquired infections in Australia--one nation, many states. J Hosp Infect. (2007) 65:174–81. doi: 10.1016/S0195-6701(07)60039-5 17540266

[19] Lou J Zhu X Xiang Z Song J Huang N Jin G . Efficacy of acellular dermal matrix in improving clinical outcomes in pediatric burns: a systematic review and meta-analysis of randomized controlled trials. J Pediatr Surg. (2025) 60:162270. doi: 10.1016/j.jpedsurg.2025.162270, 40086159

[20] Czech O Wrzeciono A Batalík L Szczepańska-Gieracha J Malicka I Rutkowski S. Virtual reality intervention as a support method during wound care and rehabilitation after burns: a systematic review and meta-analysis. Complement Ther Med. (2022) 68:102837. doi: 10.1016/j.ctim.2022.102837, 35490982

[21] Lou J Xiang Z Zhu X Song J Huang N Li J . The efficacy and safety of androgen analog oxandrolone in improving clinical outcomes in burn patients: a systematic review and meta-analysis of randomized controlled trials. Front Med. (2025) 12:1485474. doi: 10.3389/fmed.2025.1485474, 40861228 PMC12370634

[22] Sbidian E Chaimani A Garcia-Doval I Do G Hua C Mazaud C . Systemic pharmacological treatments for chronic plaque psoriasis: a network meta-analysis. Cochrane Database Syst Rev. (2017) 12:CD011535. doi: 10.1002/14651858.CD011535.pub2, 29271481 PMC6486272

[23] Lou J Xiang Z Li J Cui S Huang N Jin G . The beneficial impact of virtual reality in the burn wound care of pediatric patients: an updated systematic review and meta-analysis. Burns. (2025) 51:107623. doi: 10.1016/j.burns.2025.107623, 40763481

[24] Lou J Xiang Z Zhu X Song J Huang N Li J . Evaluating the therapeutic efficacy and safety of alginate-based dressings in burn wound and donor site wound management associated with burn surgery: a systematic review and meta-analysis of contemporary randomized controlled trials. BMC Surg. (2025) 25:215. doi: 10.1186/s12893-025-02956-z, 40380141 PMC12083019

[25] Gao Y Xu Y Liu N Fan L. Effectiveness of virtual reality intervention on reducing the pain, anxiety and fear of needle-related procedures in paediatric patients: a systematic review and meta-analysis. J Adv Nurs. (2023) 79:15–30. doi: 10.1111/jan.15473, 36330583

[26] Lou J Xiang Z Zhu X Song J Huang N Li J . Oxandrolone for burn patients: a systematic review and updated meta-analysis of randomized controlled trials from 2005 to 2025. World J Emerg Surg. (2025) 20:75. doi: 10.1186/s13017-025-00648-w, 41023744 PMC12481747

[27] Li Z Wei L Jing Y Zhang S. Effect of early nutritional support nursing intervention on postoperative recovery in patients with critical cardiac surgery: a systematic review and meta-analysis. J Perianesth Nurs. (2025). doi: 10.1016/j.jopan.2025.07.012 [Epub ahead of print]., 41452307

[28] Lou J Cui S Huang N Jin G Chen C Fan Y . Efficacy of probiotics or synbiotics in critically ill patients: a systematic review and meta-analysis. Clin Nutr ESPEN. (2024) 59:48–62. doi: 10.1016/j.clnesp.2023.11.003, 38220407

[29] Abbasi S Sigari AA Rostami S Tavakoli R Sedaghat N. The effect of LactoCare® probiotic supplementation on inflammatory markers and prognostic scores in multiple trauma patients in the ICU: a randomized controlled trial. Clin Nutr ESPEN. (2023) 55:30–7. doi: 10.1016/j.clnesp.2023.01.004 37202060

[30] Consoli MLD da Silva RS Nicoli JR Bruña-Romero O da Silva RG Generoso S d V . Randomized clinical trial: impact of oral administration of *Saccharomyces boulardii* on gene expression of intestinal cytokines in patients undergoing colon resection. J Parenter Enter Nutr. (2015) 39:715–22. doi: 10.1177/0148607115584387, 25917895

[31] Diepenhorst GMP van Ruler O Besselink MG van Santvoort HC Wijnandts PR Renooij W . Influence of prophylactic probiotics and selective decontamination on bacterial translocation in patients undergoing pancreatic surgery: a randomized controlled trial. Shock. (2011) 35:9–16. doi: 10.1097/SHK.0b013e3181ed8f17 20577144

[32] Han CM Yu JX Fu SZ. Effect of early enteral nutrition with synbiotics on plasma endotoxin levels in serious burned patients. Acta Nutr Sin. (2004) 26:66–9.

[33] Kotzampassi K Giamarellos-Bourboulis EJ Voudouris A Kazamias P Eleftheriadis E. Benefits of a synbiotic formula (Synbiotic 2000Forte) in critically ill trauma patients: early results of a randomized controlled trial. World J Surg. (2006) 30:1848–55. doi: 10.1007/s00268-005-0653-1, 16983476

[34] Kotzampassi K Stavrou G Damoraki G Georgitsi M Basdanis G Tsaousi G . A four-probiotics regimen reduces postoperative complications after colorectal surgery: a randomized, double-blind, placebo-controlled study. World J Surg. (2015) 39:2776–83. doi: 10.1007/s00268-015-3071-z, 25894405

[35] Liu Z Qin H Yang Z Xia Y Liu W Yang J . Randomised clinical trial: the effects of perioperative probiotic treatment on barrier function and post-operative infectious complications in colorectal cancer surgery—a double-blind study. Aliment Pharmacol Ther. (2011) 33:50–63. doi: 10.1111/j.1365-2036.2010.04492.x 21083585

[36] Liu Z Li C Huang MJ Tong C Zhang XW Wang L . Positive regulatory effects of perioperative probiotic treatment on postoperative liver complications after colorectal liver metastases surgery: a double-center and double-blind randomized clinical trial. BMC Gastroenterol. (2015) 15:34. doi: 10.1186/s12876-015-0260-z, 25881090 PMC4374379

[37] Lu X Han CM Yu JX Fu SZ. Preliminary comparative study on the effects of early enteral supplementation of synbiotics on severely burned patients. Chin J Burns. (2004) 20:189–92. 15447816

[38] Mangell P Thorlacius H Syk I Jernberg C Giske CG. *Lactobacillus plantarum* 299v does not reduce enteric bacteria or bacterial translocation in patients undergoing colon resection. Dig Dis Sci. (2012) 57:1915–24. doi: 10.1007/s10620-012-2102-y 22434095

[39] Rayes N Seehofer D Hansen S Boucsein K Müller AR Serke S . Early enteral supply of lactobacilli and fiber versus selective bowel decontamination: a controlled trial in liver transplant recipients. Transplantation. (2002) 74:123–7. doi: 10.1097/00007890-200207150-00021 12134110

[40] Rayes N Seehofer D Theruvath T Mogl M Langrehr JM Nussler NC . Effect of enteral nutrition and synbiotics on bacterial infection rates after pylorus-preserving pancreaticoduodenectomy: a randomized, double-blind trial. Ann Surg. (2002) 236:28–36. doi: 10.1097/01.sla.0000259442.78947.19, 17592288 PMC1899208

[41] Sadahiro S Suzuki T Tanaka A Okada K Kamata H Ozaki T . Comparison between oral antibiotics and probiotics as bowel preparation for elective colon cancer surgery to prevent infection: prospective randomized trial. Surgery. (2014) 155:493–503. doi: 10.1016/j.surg.2013.06.002, 24524389

[42] Sun Y Zhang L Li X. Effect of synbiotics on intestinal barrier function and endotoxemia in patients with severe burns. Chin J Traumatol. (2015) 18:145–8.

[43] Tan M Zhu JC Du J Zhang LM Yin HH. Effects of probiotics on serum levels of Th1/Th2 cytokine and clinical outcomes in severe traumatic brain-injured patients: a prospective randomized pilot study. Crit Care. (2011) 15:R268. doi: 10.1186/cc10468, 22136422 PMC3388628

[44] Wang H Li J Zhang L. Probiotics combined with glutamine enhance immune function and promote wound healing in patients with severe burns. Burns. (2021) 47:621–7. doi: 10.1016/j.burns.2020.10.01532839038

[45] Zhang JW Du P Gao J Yang BR Fang WJ Ying CM. Preoperative probiotics decrease postoperative infectious complications of colorectal cancer. Am J Med Sci. (2012) 343:199–205. doi: 10.1097/MAJ.0b013e318245c8b8, 22197980

[46] Zhang XW Liu ZH Huang MJ. Effect of triple viable *Bifidobacterium* on immune parameters in patients undergoing elective colon resection. Chin J Gastroenterol. (2012) 17:231–4.

[47] Zhou Y Wang L Li X. Prebiotics improve intestinal microbiota and reduce endotoxemia in pediatric burn patients: a randomized controlled trial. J Pediatr Surg. (2021) 56:1321–5. doi: 10.1016/j.jpedsurg.2021.04.012

[48] Hashmi TM Ashraf H Burhan M Zia R Ahmed M Ahmed R . Effects of dexmedetomidine on acute kidney injury and perioperative outcomes in aortic vascular surgery: a systematic review and Meta-analysis. Semin Cardiothorac Vasc Anesth. (2025) 29:291–9. doi: 10.1177/10892532251346645, 40563160

[49] Qi F Xu Y Zheng B Li Y Zhang J Liu Z . The core-shell microneedle with probiotic extracellular vesicles for infected wound healing and microbial homeostasis restoration. Small. (2024) 20:e2401551. doi: 10.1002/smll.202401551, 39109958

[50] Knackstedt R Knackstedt T Gatherwright J. The role of topical probiotics on wound healing: a review of animal and human studies. Int Wound J. (2020) 17:1687–94. doi: 10.1111/iwj.13451, 32869480 PMC7949352

[51] Abdollahpour D Homayouni-Rad A Nourizadeh R Hakimi S Mehrabi E. The effect of probiotic supplementation on episiotomy wound healing among primiparous women: a triple-blind randomized clinical trial. BMC Complement Med Ther. (2023) 23:149. doi: 10.1186/s12906-023-03980-3, 37147630 PMC10161970

[52] Twetman S Keller MK Lee L Yucel-Lindberg T Pedersen AML. Effect of probiotic lozenges containing *Lactobacillus reuteri* on oral wound healing: a pilot study. Benefic Microbes. (2018) 9:691–6. doi: 10.3920/BM2018.0003, 29726282

[53] Twetman S Pedersen AML Yucel-Lindberg T. Probiotic supplements containing *Lactobacillus reuteri* does not affect the levels of matrix metalloproteinases and interferons in oral wound healing. BMC Res Notes. (2018) 11:759. doi: 10.1186/s13104-018-3873-9, 30359300 PMC6203191

[54] Jiang L Li Q Liao H Liu H Wang Z. Enhancing agricultural productivity in dairy cow mastitis management: innovations in non-antibiotic treatment technologies. Vet Sci. (2025) 12:662. doi: 10.3390/vetsci12070662, 40711322 PMC12298687

[55] Orlando A Maqoud F Mallardi D Drago S Malerba E Chimienti G . *Lactobacillus rhamnosus* GG and *Lactobacillus paracasei* IMPC2.1 mitigate LPS-induced epithelial barrier dysfunction: a focus on autophagy regulation. Int J Mol Sci. (2025) 26:11148. doi: 10.3390/ijms262211148, 41303631 PMC12652688

[56] Zhou M Yang Y Wang S Zhang J. *Ophiopogon* polysaccharide can improve memory impairment induced by sleep deprivation in aged rats by regulating gut microbiota and inhibiting TLR4/NF-κB pathway in hippocampus. Exp Neurol. (2025) 397:115601. doi: 10.1016/j.expneurol.2025.115601, 41419172

[57] Srivastava S Mohanty B. Probiotics as an adjunct ameliorates ovarian toxicity in endotoxemic mice via modulating TLR 4/MyD88/NF-κB signalling pathway: insights from in vivo and in silico study. Reprod Sci. (2025) 32:3698–717. doi: 10.1007/s43032-025-02009-z 41145945

[58] La S Abaidullah M Li H Cui Y Liu B Shi Y. Alfalfa polysaccharide alleviates colitis by regulating intestinal microbiota and the intestinal barrier against the TLR4/MyD88/NF-κB pathway. Nutrients. (2025) 17:3001. doi: 10.3390/nu17183001, 41010526 PMC12472594

[59] Hu Y Wang Y Li X Xie Q Lyu Z. *Clostridium butyricum* JJ100 and *Lacticaseibacillus rhamnosus* LR alleviate liver injury in mice caused by continuous high-dose-alcohol exposure by protecting the intestinal barrier, rebuilding the gut microbiota and regulating AMPK and TLR4/NF-κB signaling pathways. Food Funct. (2025) 16:6687–702. doi: 10.1039/d5fo00915d, 40737044

[60] Huo Y Wang Y Ma N Guo Y Khan A Mai W. Dietary supplementation of *Lactobacillus casei* alleviates permethrin exposure-induced zebrafish testis damage through modulation of TLR4/NF-κB and AKT/Nrf2 pathways: oxidative stress, inflammation and ferroptosis. Pestic Biochem Physiol. (2025) 212:106450. doi: 10.1016/j.pestbp.2025.106450, 40500058

[61] Xie Z Zhang G Liu R Wang Y Tsapieva AN Zhang L . Heat-killed *Lacticaseibacillus paracasei* repairs lipopolysaccharide-induced intestinal epithelial barrier damage via MLCK/MLC pathway activation. Nutrients. (2023) 15:1758. doi: 10.3390/nu15071758, 37049598 PMC10097264

[62] Olimpio F da Silva JRM Vieira RP Oliveira CR Aimbire F. *Lacticaseibacillus rhamnosus* modulates the inflammatory response and the subsequent lung damage in a murine model of acute lung inflammation. Clinics (Sao Paulo, Brazil). (2022) 77:100021. doi: 10.1016/j.clinsp.2022.100021, 35303586 PMC8931357

[63] Ying M Yu Q Zheng B Wang H Wang J Chen S . Cultured *Cordyceps sinensis* polysaccharides modulate intestinal mucosal immunity and gut microbiota in cyclophosphamide-treated mice. Carbohydr Polym. (2020) 235:115957. doi: 10.1016/j.carbpol.2020.115957, 32122493

[64] Deng X Zheng C Wang S Yang R Liu Z Chen T. Treatment with a probiotic combination reduces abdominal adhesion in rats by decreasing intestinal inflammation and restoring microbial composition. Oncol Rep. (2020) 43:986–98. doi: 10.3892/or.2020.7463, 32020233

[65] Lou J Xiang Z Fan Y Song J Huang N Li J . The efficacy and safety of autologous epidermal cell suspensions for re-epithelialization of skin lesions: a systematic review and meta-analysis of randomized trials. Skin Res Technol. (2024) 30:e13820. doi: 10.1111/srt.13820, 38898373 PMC11186709

[66] Zhang W Liao Y Lou J Zhuang M Yan H Li Q . CircRNA_Maml2 promotes the proliferation and migration of intestinal epithelial cells after severe burns by regulating the miR-93-3p/FZD7/Wnt/β-catenin pathway. Burns Trauma. (2022) 10:tkac009. doi: 10.1093/burnst/tkac009, 35265724 PMC8900685

[67] Lou J Zhu X Xiang Z Fan Y Song J Huang N . The efficacy and safety of negative pressure wound therapy in paediatric burns: a systematic review and meta-analysis of randomized controlled trials. BMC Pediatr. (2024) 24:807. doi: 10.1186/s12887-024-05302-z, 39696096 PMC11653751

[68] Kanmani P Ansari A Villena J Kim H. Immunobiotics beneficially modulate TLR4 Signaling triggered by lipopolysaccharide and reduce hepatic steatosis in vitro. J Immunol Res. (2019) 2019:3876896. doi: 10.1155/2019/3876896, 31001563 PMC6437725

[69] Lou J Li J Fan Y Zhang C Huang N. Effects of virtual reality on analgesia in wound care and physical therapy for burn patients: a systematic review and meta-analysis. Pain Manag Nurs. (2024) 25:377–88. doi: 10.1016/j.pmn.2024.03.002, 38702259

[70] Peng B Huang X Xue Q Tang J Wan F Peng Y . Dexmedetomidine reduces the inflammation level and morality in adult sepsis: a systemic review and meta-analysis based on randomized controlled trials. Front Med. (2025) 12:1695924. doi: 10.3389/fmed.2025.1695924, 41195185 PMC12583032

