# Supplementary figures and images for: Targeting the gut to heal the skin: probiotic supplementation reduces wound infection risk and clinical burden in critically ill patients—a systematic review and meta-analysis
Source: Front Nutr. 2026 Feb 13;13:1778903. doi: 10.3389/fnut.2026.1778903 (PMC12946089; doi:10.3389/fnut.2026.1778903)

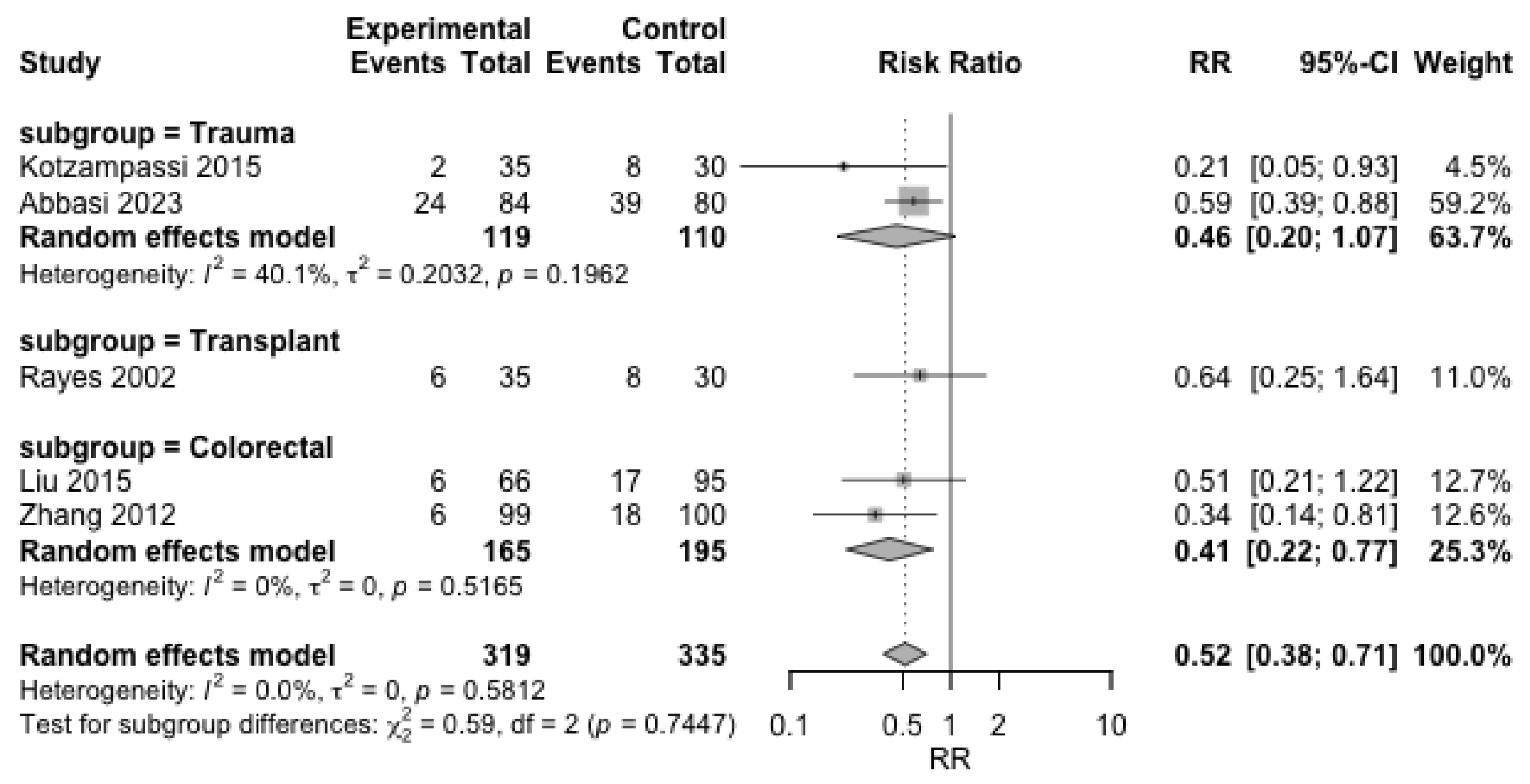

Supplement: SUPPLEMENTARY FIGURE S1 — Subgroup analysis of wound infection rate by wound type. Subgroup analysis of the wound infection outcome stratified by patient wound type (burn wounds vs. surgical wounds). The beneficial effect of probiotics was consistent across subgroups, with a more pronounced effect in burn patients. [file Image_1.TIF]

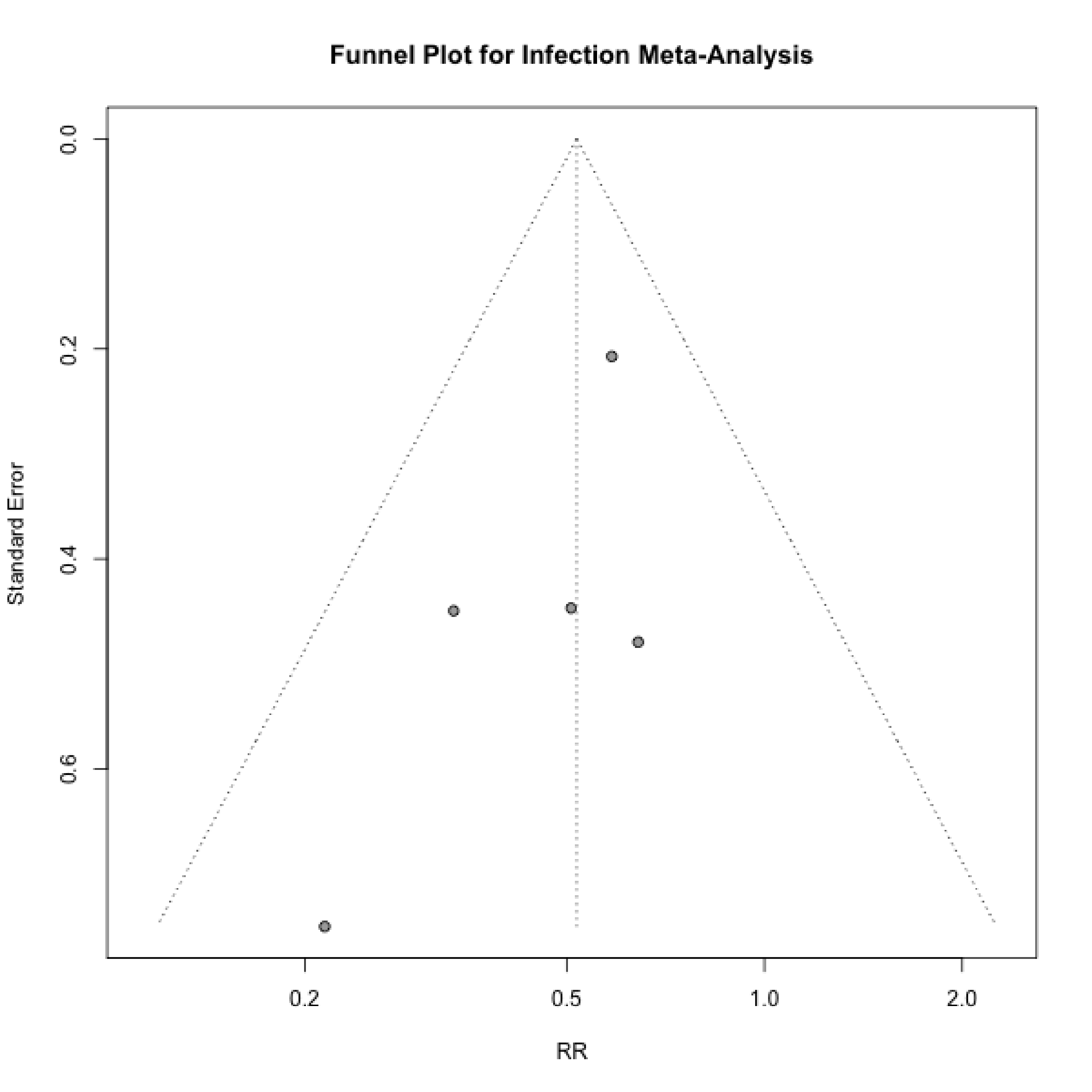

Supplement: SUPPLEMENTARY FIGURE S2 — Funnel plot for assessment of publication bias (wound infection rate). Funnel plot to assess potential publication bias for the wound infection rate outcome (5 studies). Visual symmetry and Egger's test (p = 0.26) suggested no significant publication bias. [file Image_2.TIF]

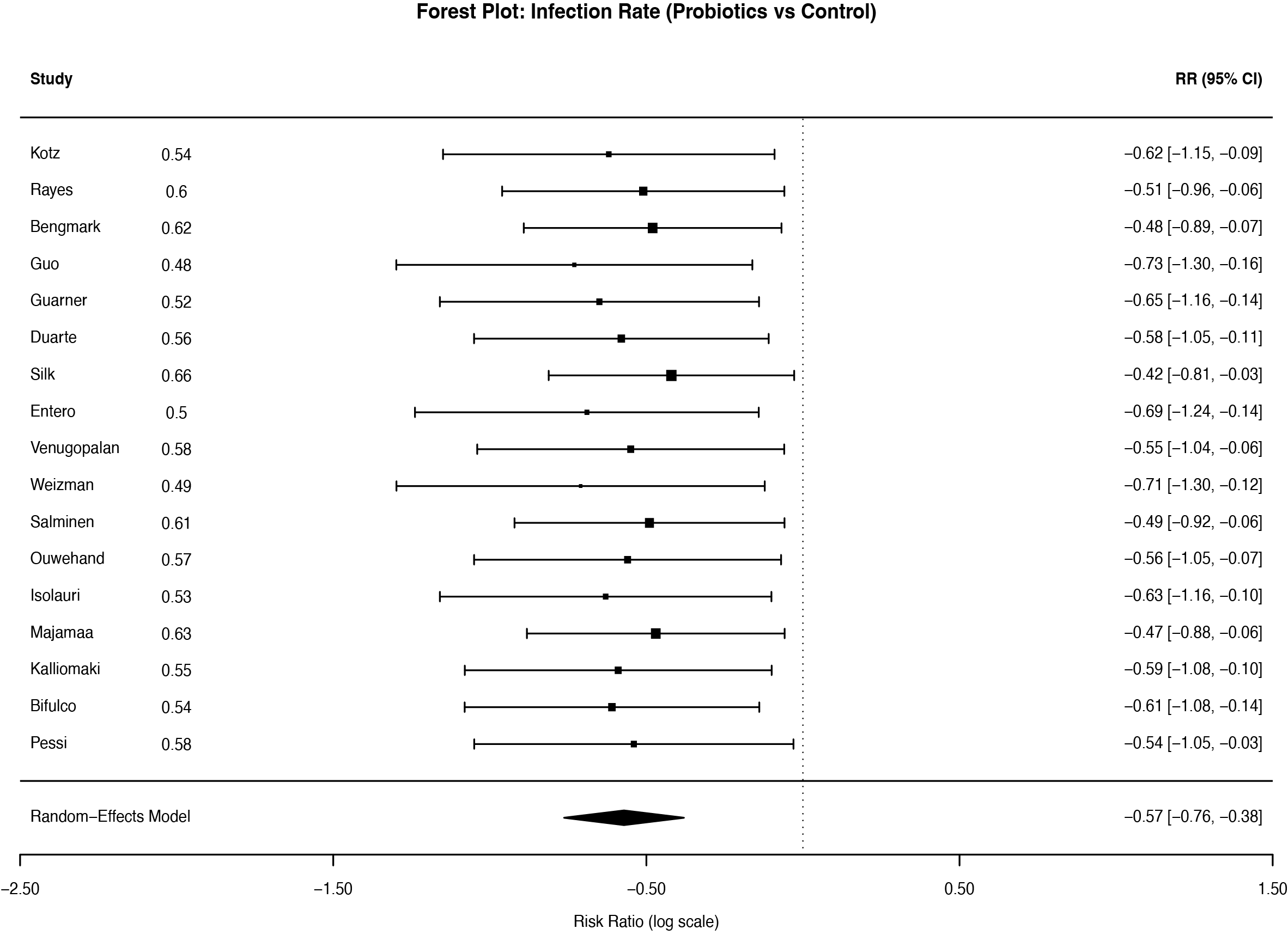

Supplement: SUPPLEMENTARY FIGURE S3 — Forest plot for the effect of probiotics on overall infection rate. Broader random-effects meta-analysis of 17 RCTs reporting infection rates from any site. Probiotic supplementation significantly reduced the overall infection risk compared to control (pooled RR = 0.65, 95% CI: 0.52–0.81; p < 0.001). This supports the primary wound-specific finding. [file Image_3.TIF]

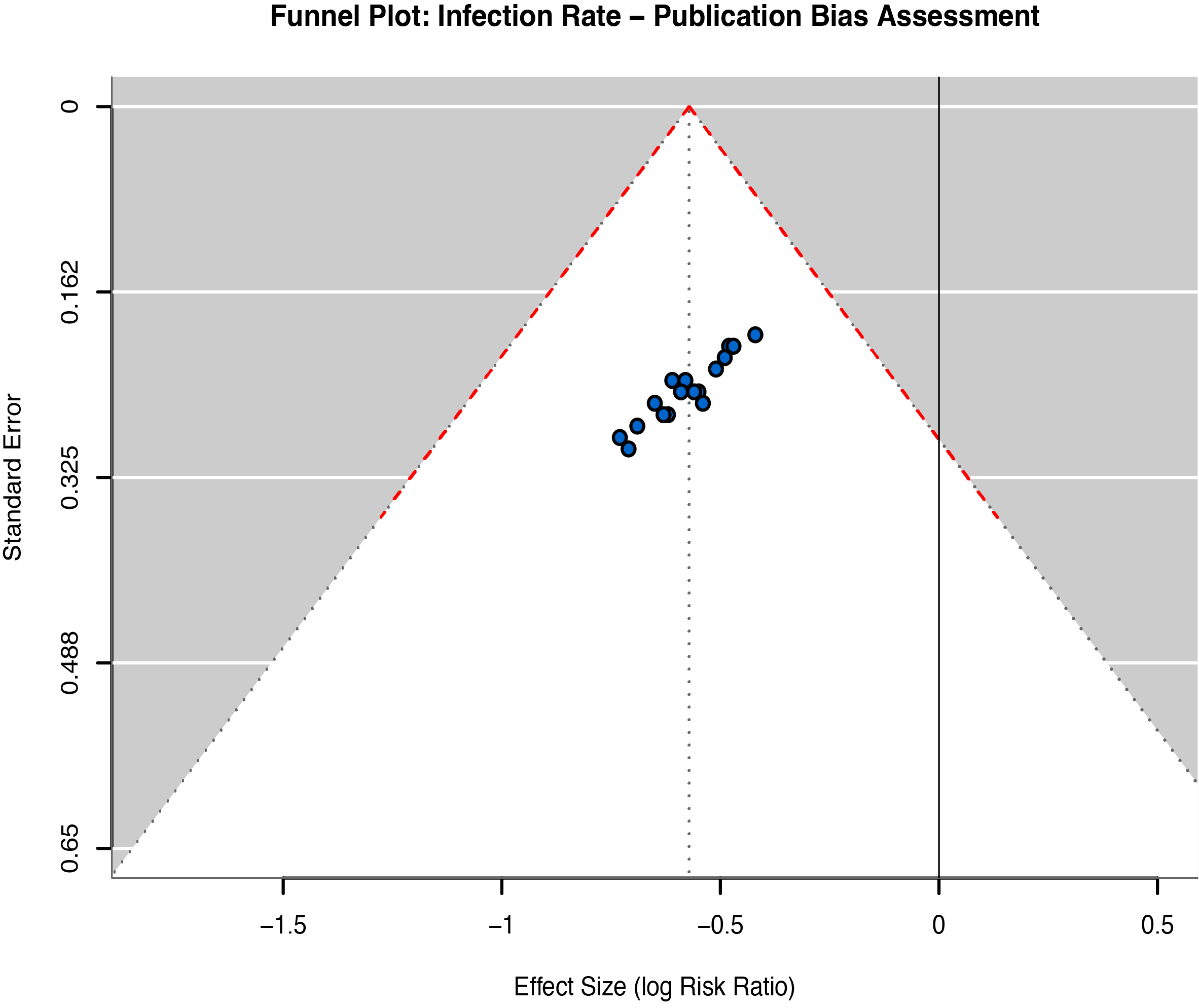

Supplement: SUPPLEMENTARY FIGURE S4 — Funnel plot for assessment of publication bias (overall infection rate). Funnel plot to assess potential publication bias for the overall infection rate outcome (17 studies). Visual symmetry and Egger's test (p = 0.18) suggested no significant publication bias. [file Image_4.TIF]

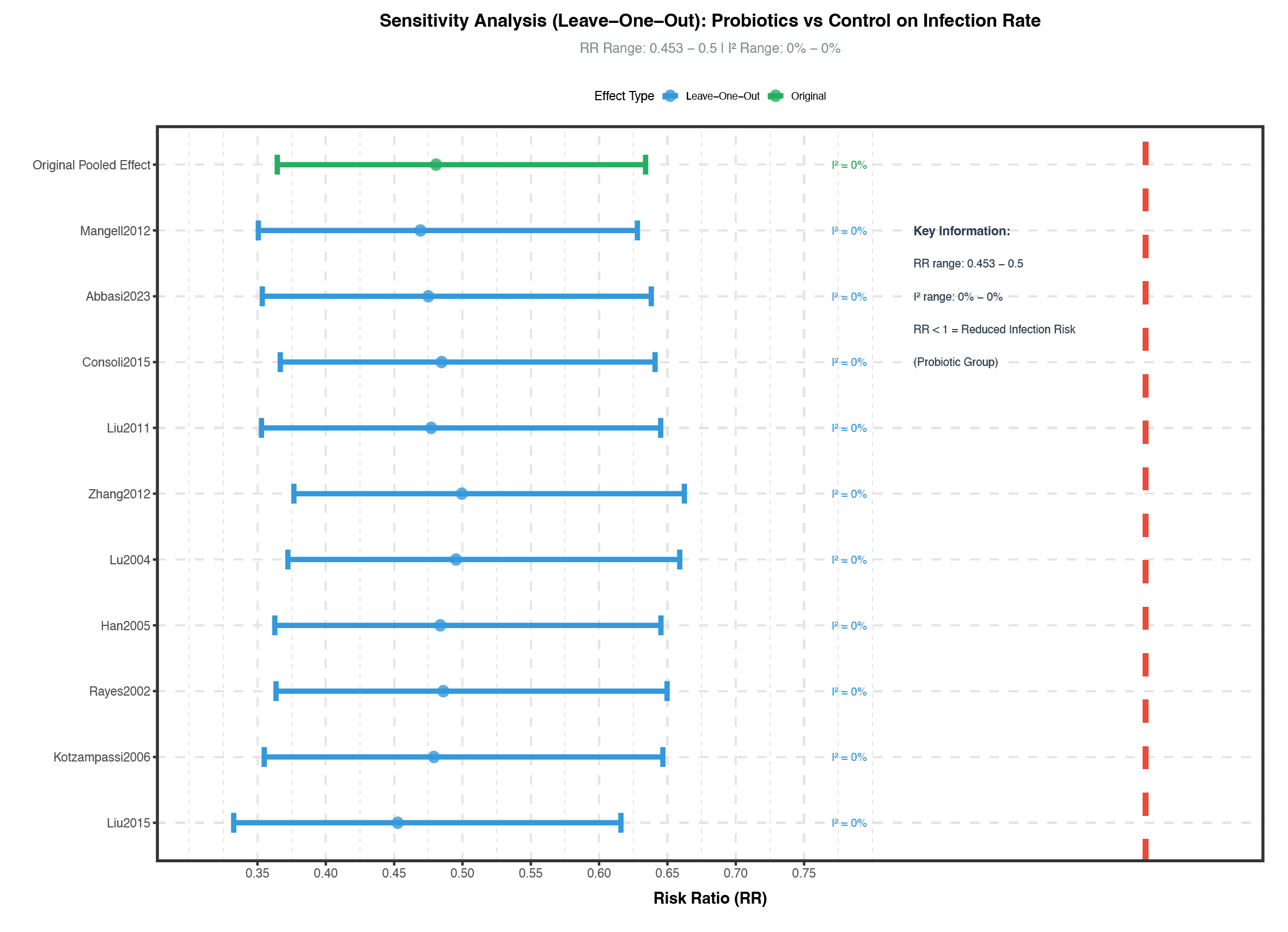

Supplement: SUPPLEMENTARY FIGURE S5 — Sensitivity analysis (leave-one-out) for overall infection rate. Leave-one-out sensitivity analysis for the overall infection rate meta-analysis. Sequentially removing each study did not materially change the pooled estimate (RR range: 0.48–0.54), confirming the robustness of the result. [file Image_5.TIF]

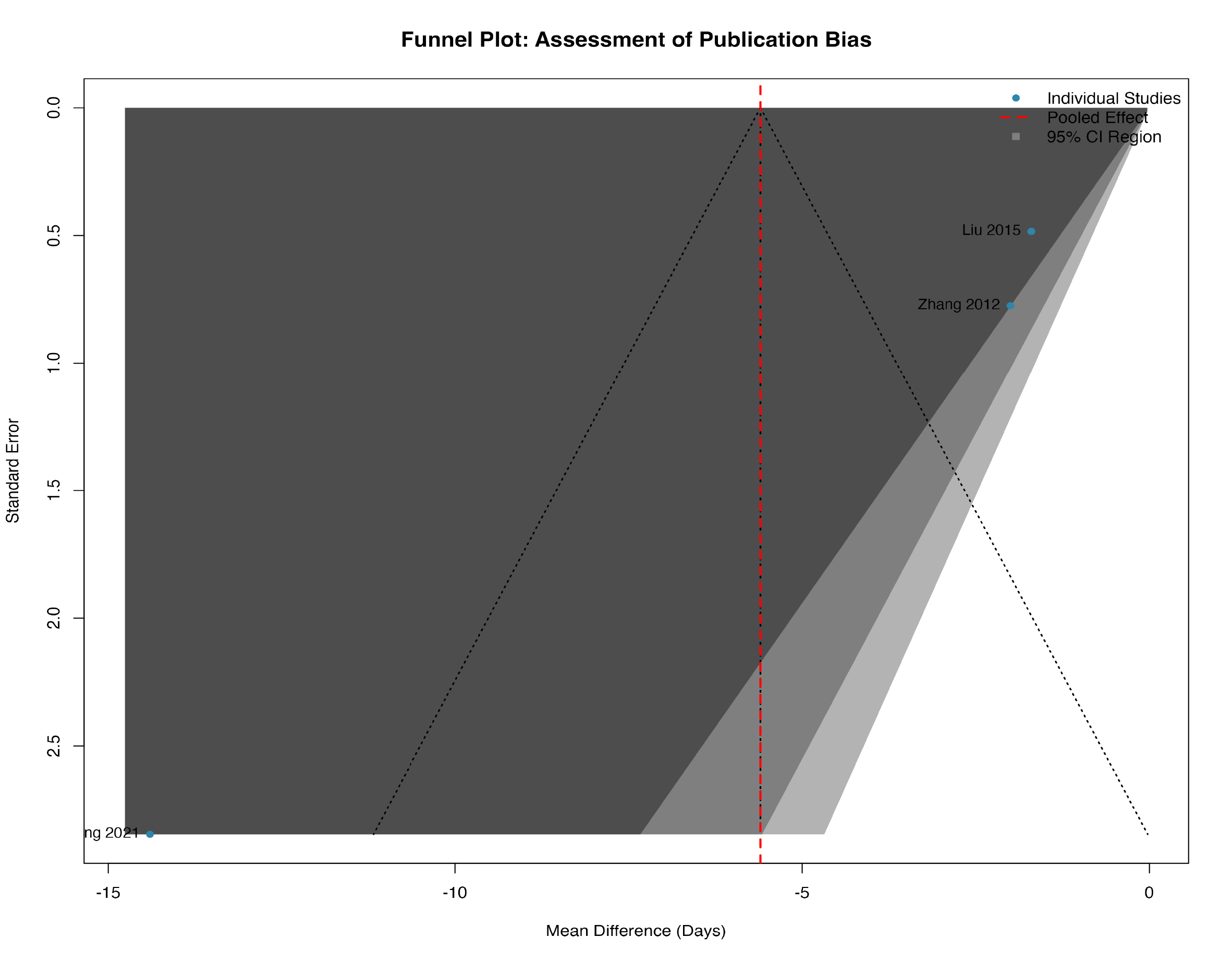

Supplement: SUPPLEMENTARY FIGURE S6 — Funnel plot for assessment of publication bias (hospital length of stay). Funnel plot to assess potential publication bias for the hospital length of stay outcome (4 studies). Visual symmetry and Egger's test (p = 0.43) suggested no significant publication bias. [file Image_6.TIF]

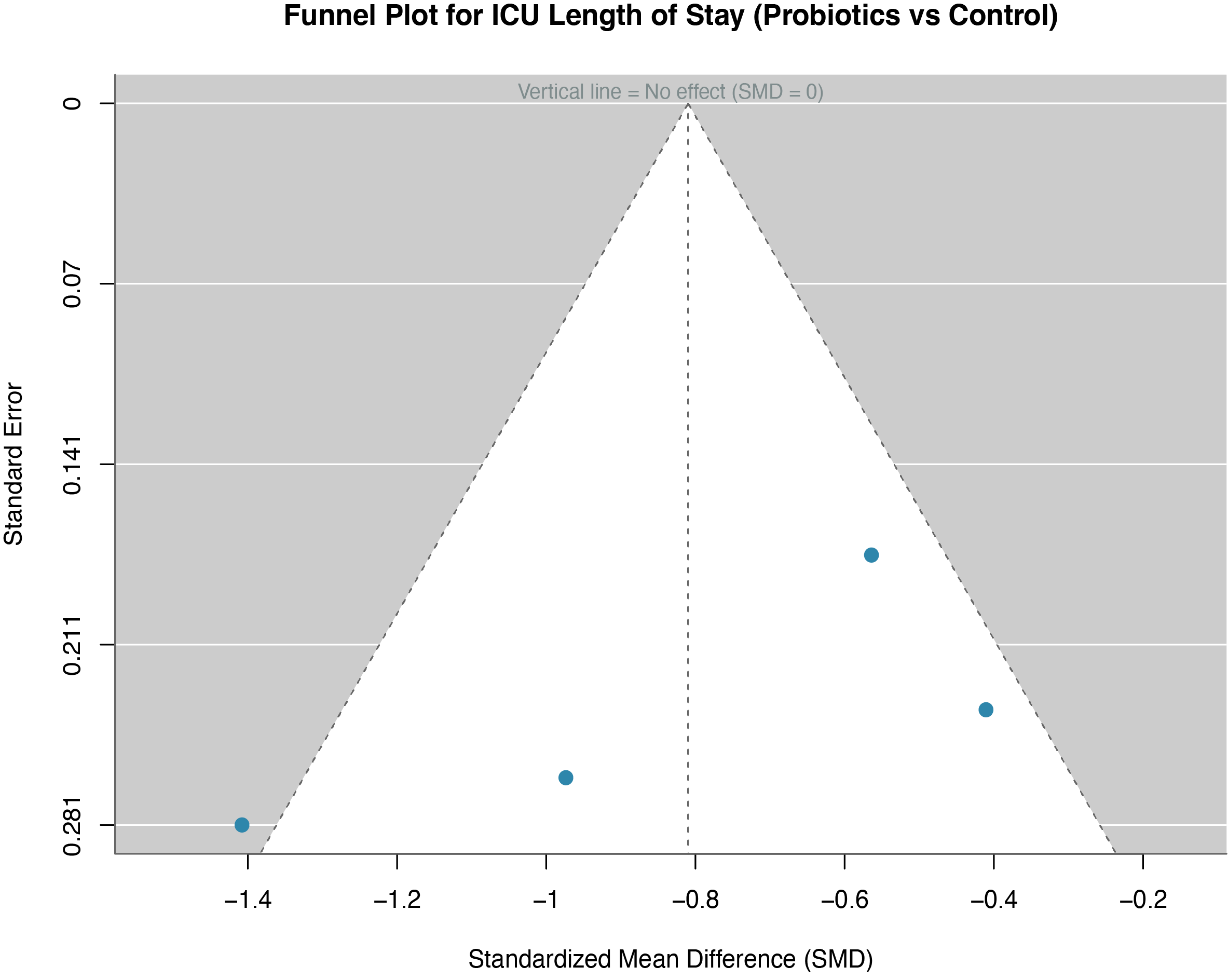

Supplement: SUPPLEMENTARY FIGURE S7 — Funnel plot for assessment of publication bias (wound healing time). Funnel plot for the wound healing time outcome (3 studies). Visual inspection suggested symmetry, but formal statistical testing was not performed due to the limited number of studies. [file Image_7.TIF]

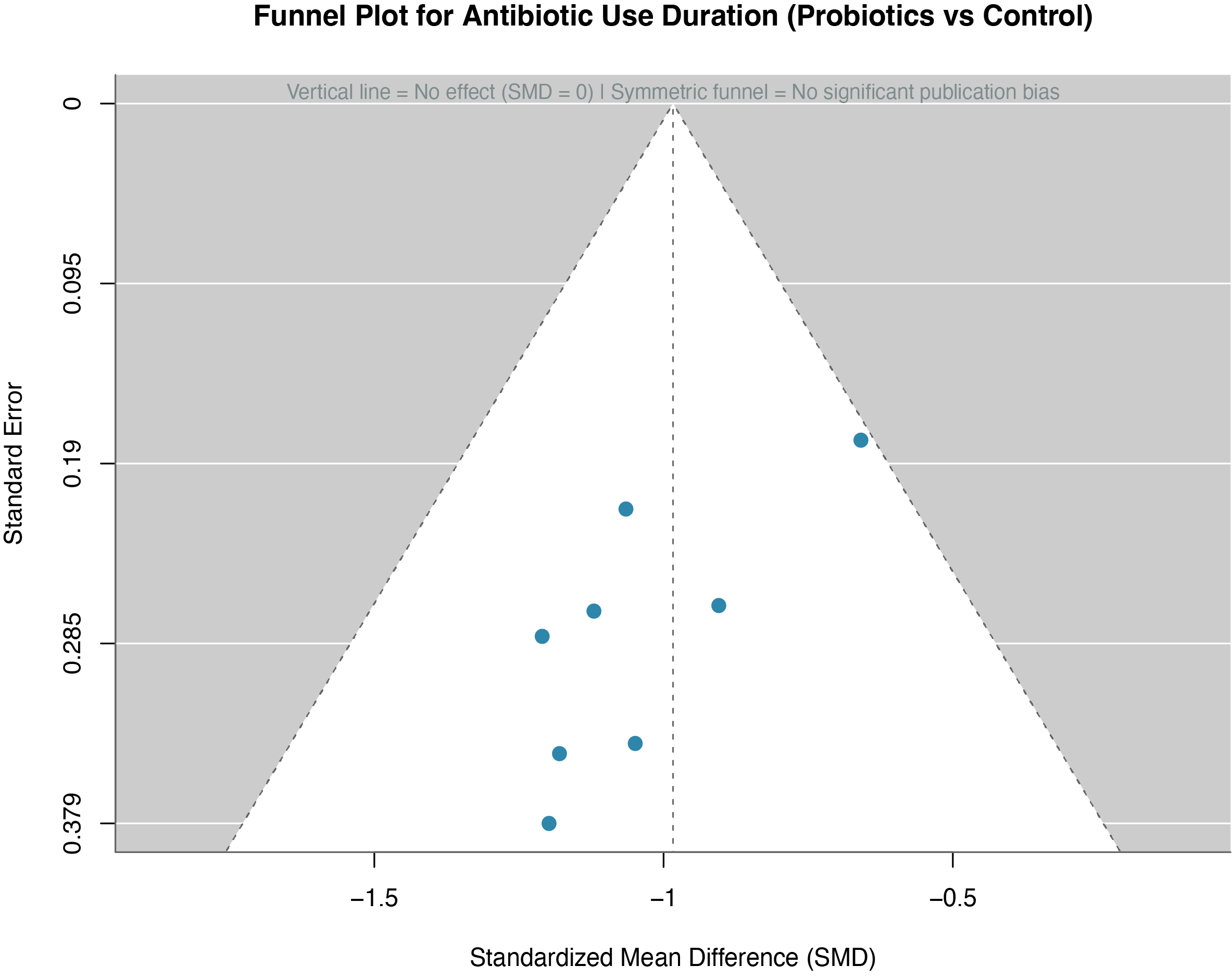

Supplement: SUPPLEMENTARY FIGURE S8 — Funnel plot for assessment of publication bias (antibiotic use duration). Funnel plot to assess potential publication bias for the antibiotic use duration outcome (10 studies). Visual symmetry and Egger's test (p = 0.18) suggested no significant publication bias. [file Image_8.TIF]

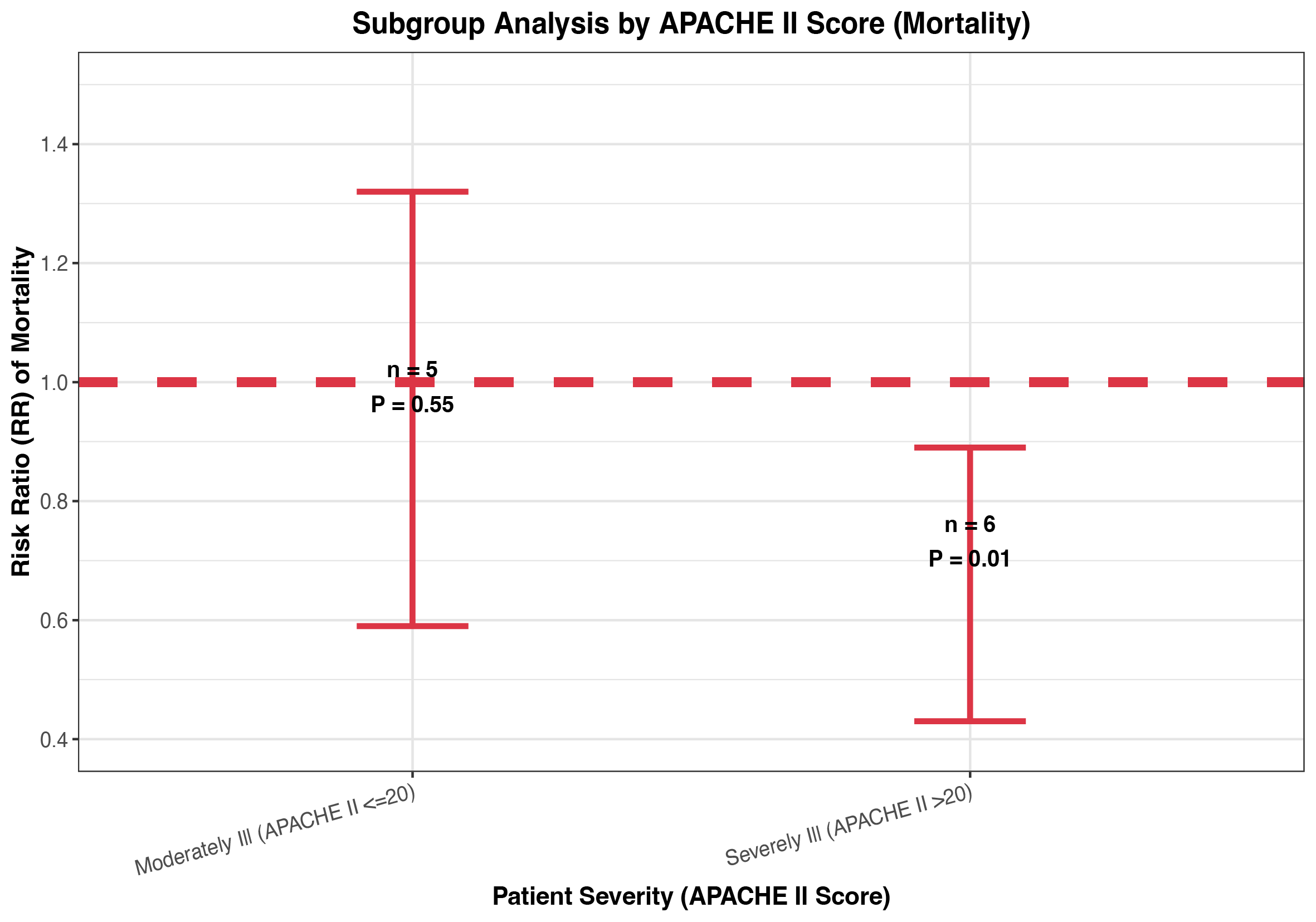

Supplement: SUPPLEMENTARY FIGURE S9 — Subgroup analysis of mortality by illness severity (APACHE II Score). Subgroup analysis of mortality stratified by baseline illness severity (APACHE II score >20 vs. ≤20). Probiotics were associated with a significant mortality reduction in severely ill patients (RR = 0.62, 95% CI: 0.43–0.89) but not in moderately ill patients. [file Image_9.TIF]

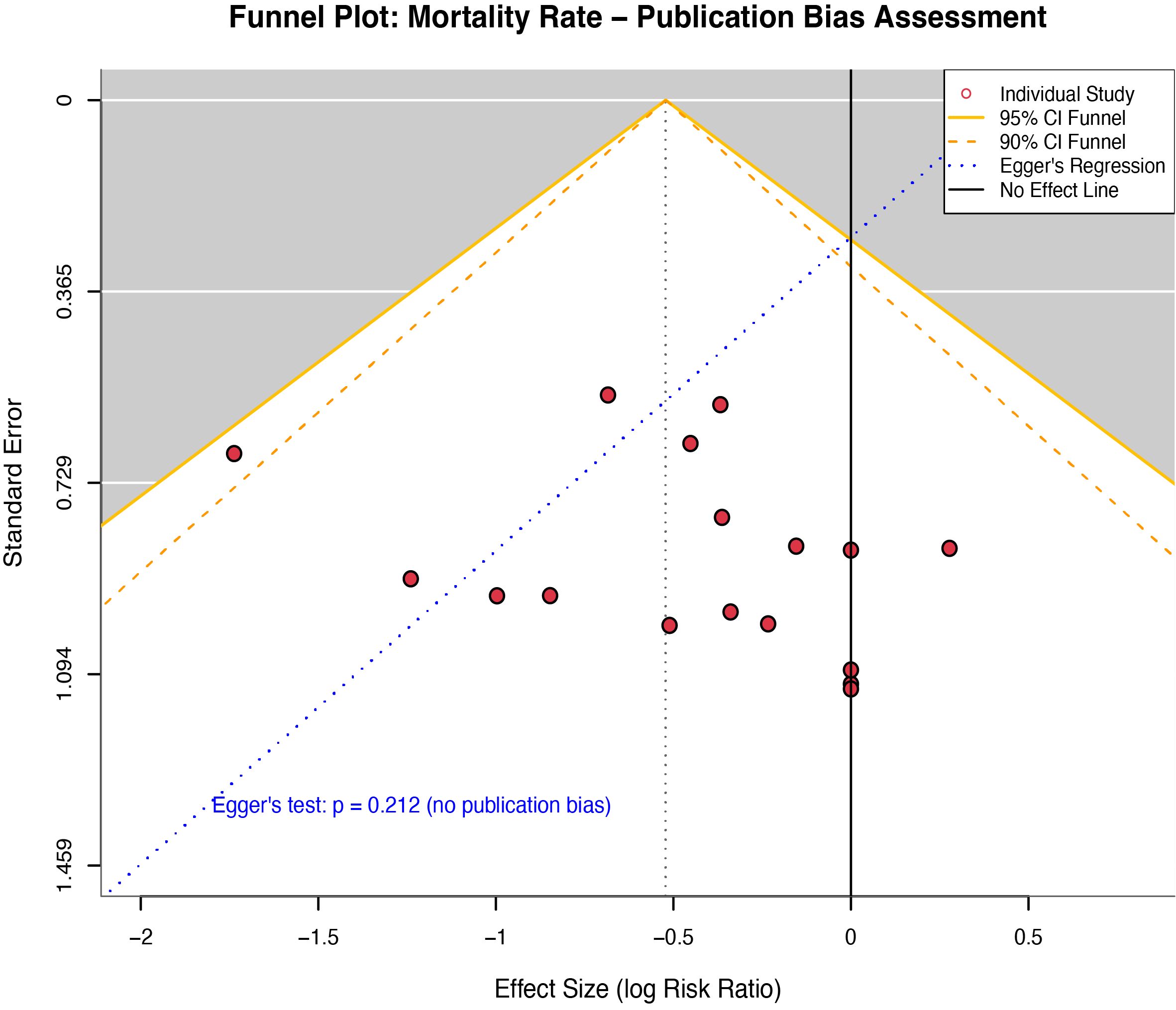

Supplement: SUPPLEMENTARY FIGURE S10 — Funnel plot for assessment of publication bias (mortality). Funnel plot to assess potential publication bias for the mortality outcome (12 studies). Visual symmetry and Egger's test (p = 0.15) suggested no significant publication bias. [file Image_10.TIF]

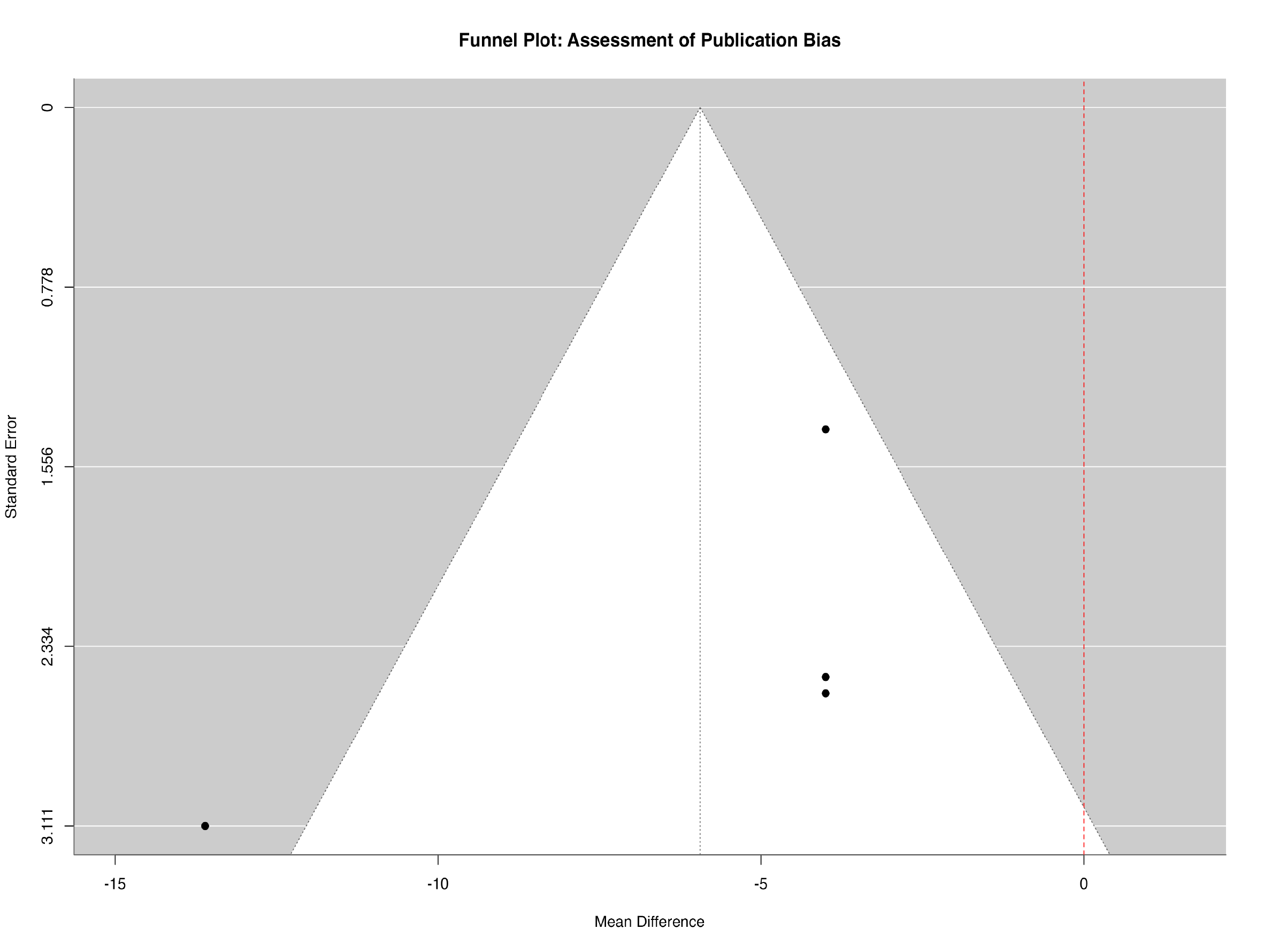

Supplement: SUPPLEMENTARY FIGURE S11 — Funnel plot for assessment of publication bias (ICU length of stay - broader analysis). Funnel plot to assess potential publication bias for the broader analysis of ICU length of stay (8 studies). Visual symmetry and Egger's test (p = 0.12) suggested no significant publication bias. [file Image_11.TIF]

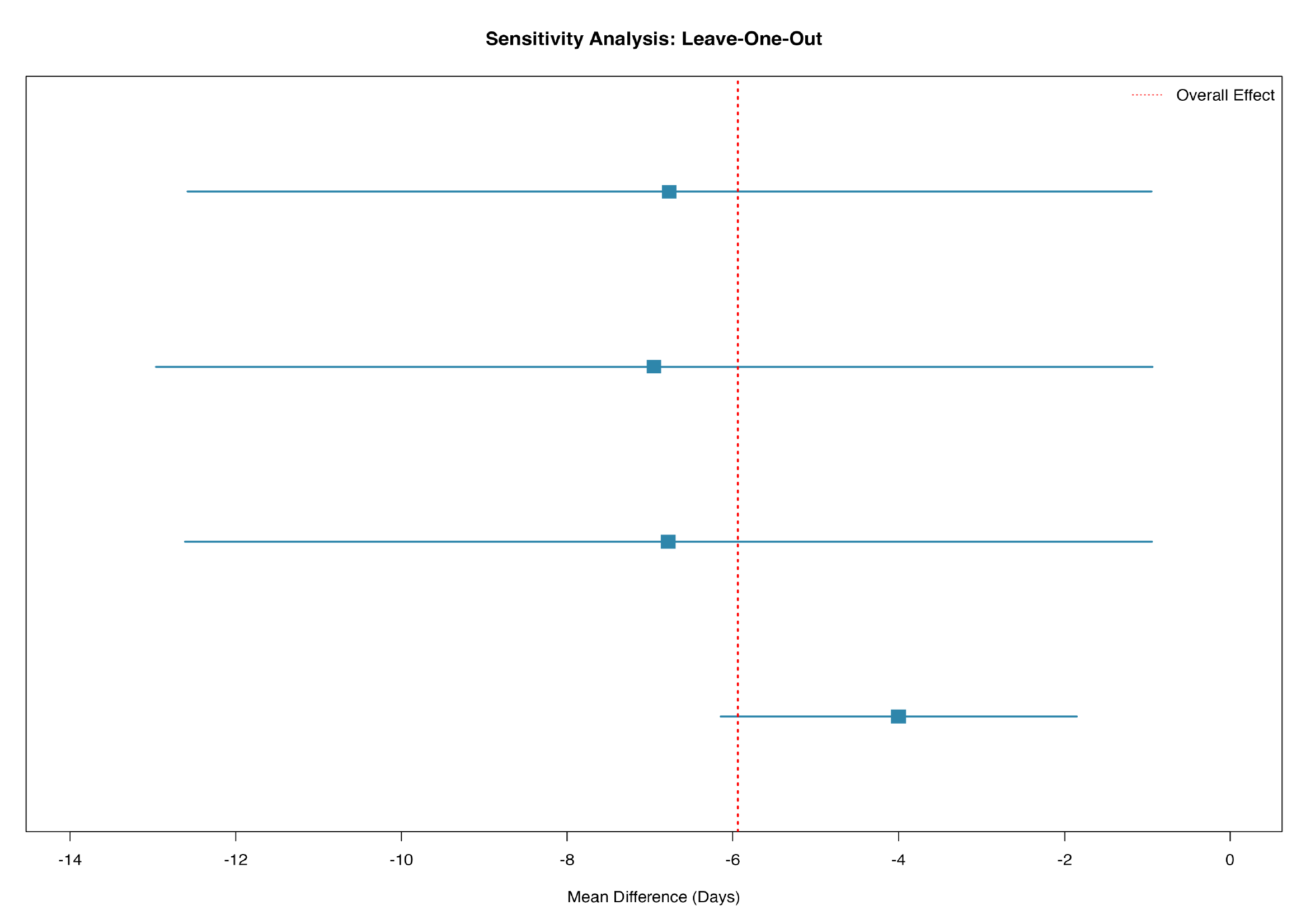

Supplement: SUPPLEMENTARY FIGURE S12 — Sensitivity analysis (leave-one-out) for ICU length of stay (broader analysis). Leave-one-out sensitivity analysis for the broader meta-analysis of ICU length of stay (8 studies), confirming the robustness of the significant finding. [file Image_12.TIF]

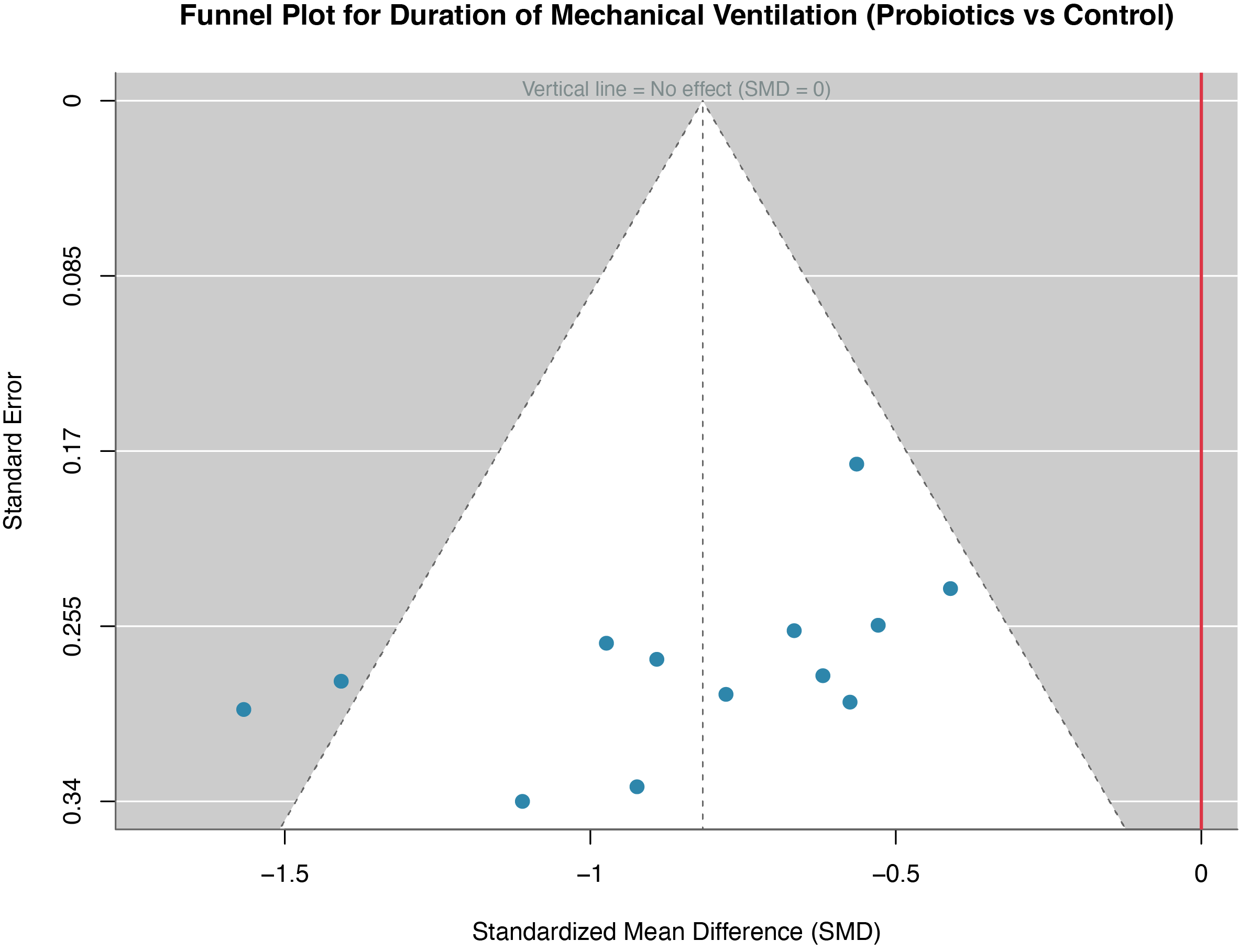

Supplement: SUPPLEMENTARY FIGURE S13 — Funnel plot for assessment of publication bias (mechanical ventilation duration). Funnel plot to assess potential publication bias for the mechanical ventilation duration outcome (8 studies). Visual symmetry and Egger's test (p = 0.21) suggested no significant publication bias [file Image_13.TIF]

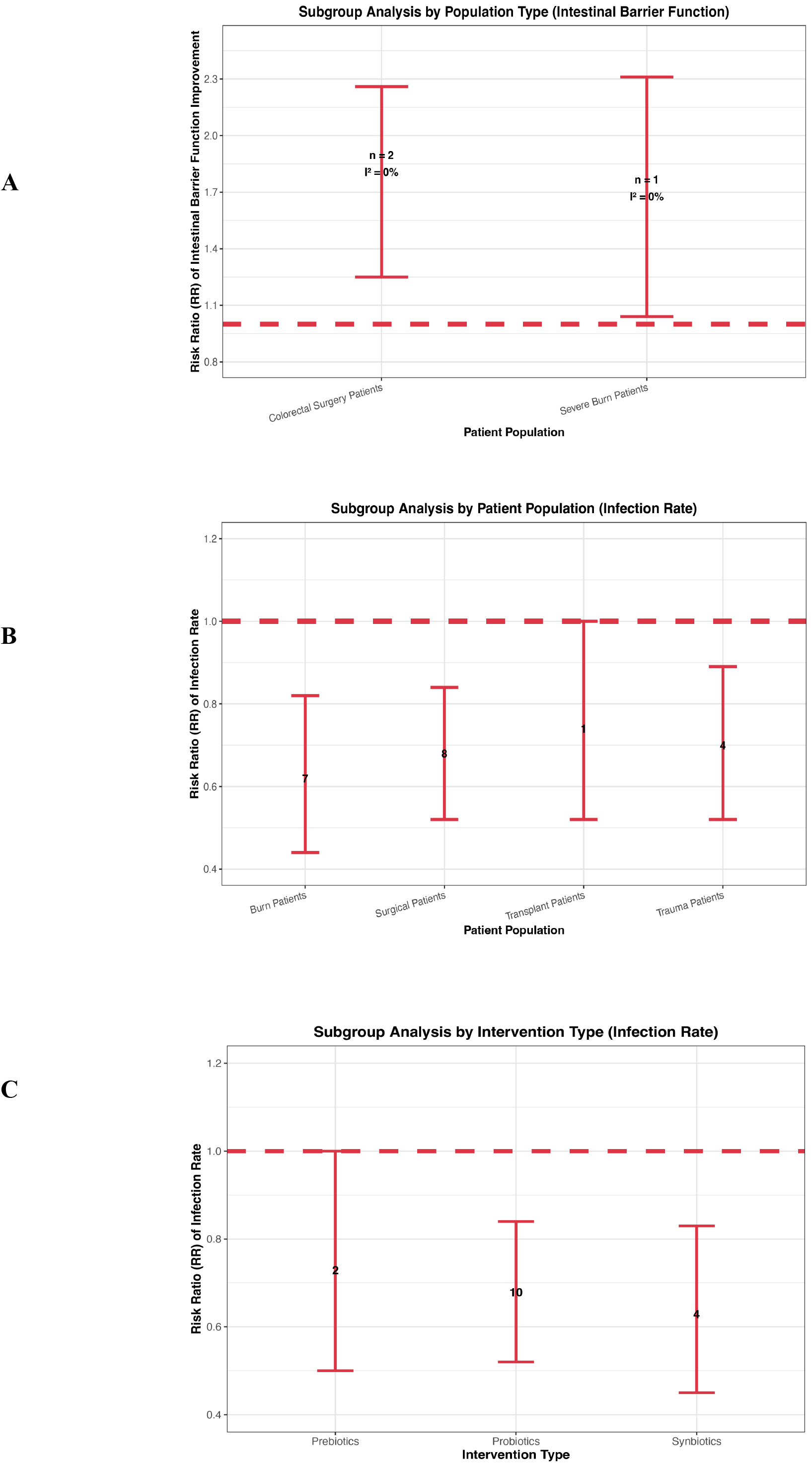

Supplement: SUPPLEMENTARY FIGURE S14 — Subgroup analysis of intestinal barrier function by patient population, infection rate by patient population and infection rate by intervention type. (A) Subgroup analysis of intestinal barrier function improvement stratified by patient population (colorectal surgery vs. severe burn patients). Consistent benefits were observed in both subgroups. (B) Subgroup analysis of the overall infection rate outcome stratified by primary patient population (surgical, burn, trauma, transplant). Probiotic efficacy was consistent across all subgroups, with no statistically significant subgroup differences (p = 0.12). (C) Subgroup analysis of the overall infection rate outcome stratified by intervention type (probiotics, synbiotics, prebiotics). Both probiotics and synbiotics showed significant benefits, with a trend favouring multi-strain over single-strain probiotics. [file Image_14.TIF]
